# Combined analysis of PS-InSAR and hypsometry integral (HI) for comparing seismic vulnerability and assessment of various regions of Pakistan

**DOI:** 10.1038/s41598-022-26159-1

**Published:** 2022-12-27

**Authors:** Uqba Ramzan, Hong Fan, Hafsa Aeman, Muhammad Ali, Mohammed A. A. Al-qaness

**Affiliations:** 1 State Key Laboratory of Information Engineering in Surveying, Mapping and Remote Sensing, Wuhan University, Wuhan, China; 2 Dipartimento di Ingegneria, Università degli Studi di Napoli Parthenope, Naples, Italy; 3 College of Physics and Electronic Information Engineering, Zhejiang Normal University, Jinhua, China

**Keywords:** Natural hazards, Solid Earth sciences, Risk factors

## Abstract

InSAR-based deformation analysis and the geomorphic hypsometric integral (HI) technique are powerful tools for assessing the susceptibility and comparison of seismic sites to earthquakes. Therefore, this paper mainly focuses on surface deformation analysis associated with the Mw 5.0 earthquake (2019) in Mach and Quetta, Balochistan, Pakistan. Sentinel-1 IW data was used to perform PS-InSAR time series analysis. SRTM DEM of 30 m spatial resolution was utilized for the geomorphic Hypsometry Integral (HI) method. The obtained results of the Interferogram indicate the changes in velocity and vertical displacement during pre-seismic, co-seismic, and post-seismic activity. Integral values were calculated using Hypsometry curves delineating the future probability and comparison of vulnerable seismological sites in Mach, Quetta, Ghazaband, Chamman and surroundings of Balochistan region. The combined results of HI and PS-InSAR revealed that Mach and Quetta regions are in between two lines known as the mature stages. Class 1_moderate (0.35 ≤ HI ≤ 0.52); with an integral value of HI_Mach_ = 0.398 and HI_Quetta_ = 0.435 with a modest seismic forthcoming rate in future and susceptible to both erosion/uplifting with a vertical displacement rate more than existing ± 55 mm/year. Class 2_high (HI ˃ 0.53) with the younger and more tectonically active region surrounded by Chaman fault, which possesses a future susceptible tendency towards subsidence more than an existing velocity rate ~ 8 mm/year and Ghazaband fault towards uplifting more than 5–6 mm/year. No region of the study area was found at Monadnock: class 3_Low (HI ˂ 0.35) stabilized condition, all sites are unstable and tectonically active. Therefore, obtained results through combined PS-InSAR and HI techniques can be used for the identification of most vulnerable seismic sites and can ascertain future safe metropolitan planning.

## Introduction

Many studies have been carried out to estimate land subsidence caused by natural processes such as tectonic activities, glacial movement, landslides monitoring and anthropogenic activities, such as the extensive withdrawal of underground natural resources using Interferometric Synthetic Aperture Radar (InSAR) techniques. Appropriate measures for monitoring land deformation can proactively address future challenges about land subsidence^1^. Compared to the space coverage of measurements points in levelling procedures (including GPS), InSAR has the advantage that the observations are acquired with almost complete coverage^2^.

Many modern methods have been developed for seismic vulnerability assessment. A machine-learning framework assesses the seismic hazard safety of reinforced concrete buildings. In the framework, the damage classification technique, and the efficacy of the Machine Learning (ML) method in damage prediction via a Support Vector Machine Type of the Paper (Article, Review, Communication, etc.) (SVM) model are discovered^3^. The hierarchical type-2 fuzzy logic model is developed to expand the quick assessment of earthquake risk safety of prevailing buildings. A novel framework for earthquake vulnerability evaluation of buildings via Rapid Visual Screening (RVS) is proposed using type-2 fuzzy^4^.

This study attempts to evaluate time-series surface displacement with a combined approach using PS-InSAR and Digital Elevation Model (DEM)-derived geomorphic Hypsometry Integral (HI) method^2^. This research mainly emphasis on two cities (Quetta and Mach) of Pakistan. The study is associated with Mw 5.0 with a 10 km depth earthquake stoke on 16 March 2019. It identifies the future seismic tendency and highlights the surface displacement in the earthquake region^5^.

Many superficial events are common with less than 15 km depression in Balochistan, Pakistan region because of predominant transform tectonics events of Chaman and Ghazaband fault. This region is seismic concerning stress accumulation with a complex fault system, making it a more complicated hazardous fault system. The area has strong antiquity of seismicity with some overwhelming and devastating earthquakes activities, including the areas of Quetta city 1935 earthquake (Mw 7.7). Furthermore, as the result of east–west compression caused by the depression of the Indian Plate to the Eurasian Plate, the Mw 7.3 earthquake was recorded in the region of Mach in 1931, which resulted in huge local uplift on a press flat line over the Bolan Pass in the northern Kirthar Range^6^.

Combination of different techniques have been previously utilized for land subsidence^7–10^ and spatial–temporal analysis^11–13^ of different areas. To monitor the surface deformation of various regions grouping of GNSS-InSAR^9^, GPS-InSAR^10,11^, Wavelet Transforms-InSAR^8^ were utilized previously. We used the Permanent Scatterer Interferometric Synthetic Aperture Radar (PS-InSAR) based time-series displacement analysis and Hypsometry Integral (HI) technique on evaluating the seismic landscape characteristics. Firstly, we analysed the Sentinel-1 based Single Interferogram processing. Secondly, we combined the yield of Hypsometry Integral values and deformation results and delineated the future probability of seismological vulnerable sites^2,5^.

The main tectonic assemblies of Balochistan include the Salt Range, chain of Sulaiman Lobe and Sulaiman Range, Ghazaband fault, Chaman Fault, and Makran ranges^14^. These areas demonstrate the highly diffusely deforming zone with high levels of seismic activity with a relative motion of Indian and Eurasian plates over both seismic and aseismic deformation slips. The eastern part of Balochistan’s fold and thrust belt is the stretch of transpressional and oblique collision between Pakistan and India and the chunk of Afghanistan^15^.

The Chaman fault is one of the energetic and active fault systems 650-km long and is considered the most activist zone with moderate to large earthquake activities^16,17^. Particularly in the area of the Chaman Fault, the large strike-slip faults are also accompanying the other thrust and folds, which makes the structural setting more susceptible to earthquakes because they are colliding at an oblique angle to the trends of the folds^18^. As both the Indian and Eurasian continental blocks are converged, and the corresponded island arcs that had been merged into the Eurasian Plate, the deposition of sedimentary were formed along the side edge of the Indian Plate, which ultimately formed a succession of fold-and-thrust belts^6,19–21^. Likewise, due to the motion of these plates number of earthquakes that occur on the Chaman Fault results from north–south to east and southwest strike-slip fault motion. In the last forty years, significant events have occurred within the range of 200 kms (120 mi), including the Mw 6.1 earthquake in July 1990^21^.

In this paper, we concisely indicate the relevant studies of Sentinel-1 to identify the deformation caused by the earthquake. Further, a GIS-based hypsometric integral approach was used to study the lithology-based seismic vulnerable sites and case study location and obtained datasets. This paper mainly focuses on PS-InSAR Derived Displacement analysis using Interferogram processing, calculation of Hypsometry Integral values for seismic vulnerability, and combination of time-series surface deformation analysis and Hypsometry Integral values for Seismic susceptibility to indicate the future tendency of seismic vulnerability sites. This is a unique study discussing PS-InSAR and HI combine analysis, which had never been done before in Pakistan. For the evaluation of results, the probability of the total earthquake is identified from the past year's earthquake data obtained from United States Geological Survey (USGS) and Environmental Systems Research Institute (ESRI) with Spatio-temporal magnitude values.

## Study area

The region of Balochistan has experienced many earthquakes in the past years with both small and high magnitudes. The most severe earthquake’s magnitude was observed near Sharigh with an Mw 6.8 on 24 August 1931. Also, it was followed by the major city of Balochistan Mach, Mw 7.3 (27 August 1931), and these earthquakes triggered severe destruction and loss of lives since the significance in economic and cultural life is high in that region and relatively due to the great seismic activity of these regions. The Mach and Quetta city were selected as the target region for the earthquake displacement analysis and evaluated the seismic landforms characteristics. As the deformation related to uplifting and erosion continues in this region, potentially moderate-to-large magnitude earthquakes are common and cause severe damage. The variety of deadly and destructive hazards associated with earthquakes impact a severe threat to renovation efforts and the economic development and growth of this region. Moreover, the Arabian Plate has caused subduction underneath the Eurasian Plate in this region with a 4 cm/year total rate. It is also linked with a sedimentary accretionary chunk of deposits.

**Figure 1:**
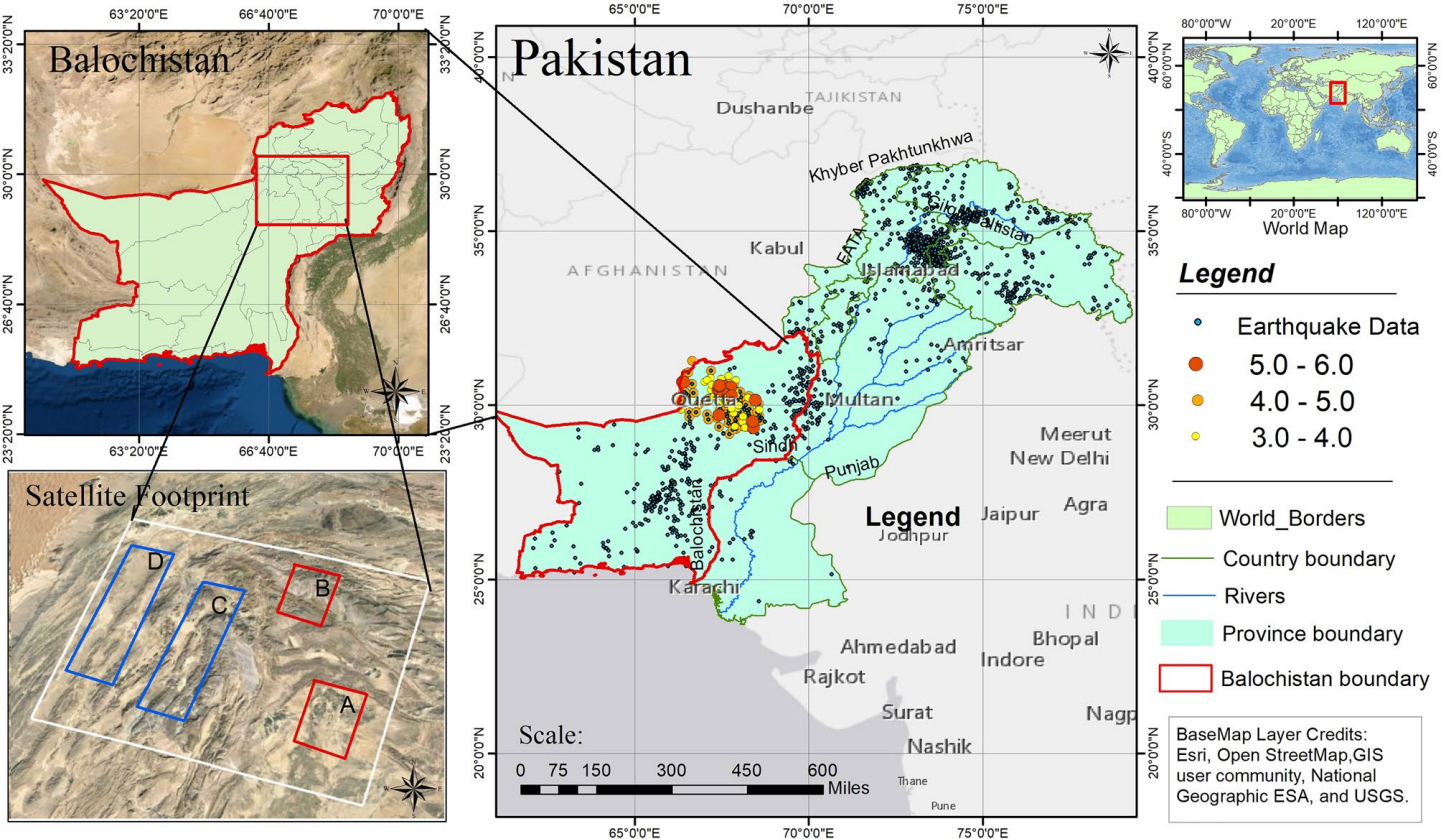
Study area map with highlighted red Provincial boundary of Balochistan, Pakistan along with earthquakes data archived from (USGS); red and blue rectangles A, B, C, D is indicating Satellite footprint of sites Mach, Quetta, Ghazaband and Chaman respectively. © Used Open Street Map by ESRI platform ©National Geographic ESA ©USGS.

The study area in Fig. 1 demonstrates the variable style of deformation (erosion and uplifting) which provides discernment into the kinematics of seismic landscape and describe a framework that can help to constrain deformational and displacement trend along the Chaman fault belt that extends 900 km to the north-eastern edge of the Makran accretionary in the south^22^.

## Materials and methods

### Datasets

PS-InSAR based time series analysis were carried out to monitor the earthquake induced deformation. We have used 34 images of Sentinel-1 IW data ranging March 2018 to May 2019 for pre, co, and post-seismic events, respectively are listed in Table 1. The IW-Single Look Complex product has an advantage as it moderates the signal-to-noise ratio (SNR) and scalloping effect, resulting in a uniform image quality^23^. As the sentinel-1 acquires at C-band products brings some significant advantages concerning other sensors with open accessibility and with a revisit time of 12 days^24^. The sentinel-1 acquires at C-band products brings some significant advantages concerning other sensors with open accessibility and a revisit time of 6 days. Sentinel-1 works under a pre-determined operational mode system to produce long-term consistency in data archives built especially for the long-term time series analysis. It is also one of those first five missions that European Space Agency (ESA) is emerging for Copernicus’s initiative, an open data source providing free SAR-Sentinel-1 datasets^25^. SARPROZ and MATLAB platforms were used for the processing of SAR data for the ground deformation analysis and the InSAR time-series analysis. Shuttle Radar Topography Mission (SRTM) dataset with the resolution of 1 arc-second (approximately 30 m) was used for the elevation profiles analysis and the hypsometric curves extraction in affected area. MATLAB and ArcGIS Package (ArcMap for 2D mapping and ArcScene for 3D mapping) were further used for the processing of DEM-based watersheds analysis and the geomorphic hypsometry integral curves values extraction. For the approximation of integration values, the statistical analysis from the hypsometric curves were retrieved through the MATLAB platform. For the Topographic Elevation Profiles analysis and the visualization of landscapes, Google Earth Pro was used for the extraction of elevation profiles. The Landsat 8 data combined with SRTM were used for the 3D seismic topographic site indication. The earthquakes data used in this research paper was retrieved from the United States Geological Survey (USGS PDE) and Pakistan Meteorological Department (PMD) earthquake data catalogues and ESRI Spatio-temporal magnitude values in Table 1. Sentinel-1 wide swath datasets acquisition from 5 March 2018 to 23 May 2019 of (Pre, co, and post) seismic event along with track, temporal and baseline information.

**Table 1:** It represents the acquisition dates of the images with temporal and baseline information.

Acquisition Dates	Track	Baseline (m)	Temporal Baseline	Acquisition dates	Track	Baseline (m)	Temporal baseline
05-Mar-18	Descending	11.33	− 204	19-Oct-18	Descending	− 17.21	24
17-Mar-18		− 33.64	− 192	31-Oct-18		− 20.66	36.0
29-Mar-18		− 3.84	− 180	12-Nov-18		− 61.94	48.0
10-Apr-18		46.76	− 168	18-Dec-18		63.21	83.9
05-May-18		33.33	− 144	30-Dec-18		− 6.07	95.9
16-May-18		− 48.75	− 132	11-Jan-19		− 0.19	107
28-May-18		− 8.547	− 120	23-Jan-19		− 78.92	119.9
09-Jun-18		30.4	− 108	04-Feb-19		7.19	131.9
16-Jul-18		78.85	− 84	16-Feb-19		7.66	143.9
15-Jul-18		6.45	− 72	28-Feb-19		− 26.74	155
27-Jul-18		17.81	− 60	12-Mar-19		3.62	167
08-Aug-18		6.79	− 48	24-Mar-19		− 51.2	179
20-Aug-18		52.52	− 36	05-Apr-19		− 89.84	191
01-Sep-18		− 23.99	− 24	17-Apr-19		− 83.11	203
13-Sep-18		16.56	− 12	29-Apr-19		− 64.6	215
25-Sep-18		0	0	11-May-19		84.39	227
07-Oct-18		19.42	12	23-May-19		− 25.55	239

The temporal baseline (day) is calculated in Table 1 to master image (25 September 2018), and the acquisitions permit a choice of pair images with the suitable baseline information and time interval.

### Methods

**Figure 2:**
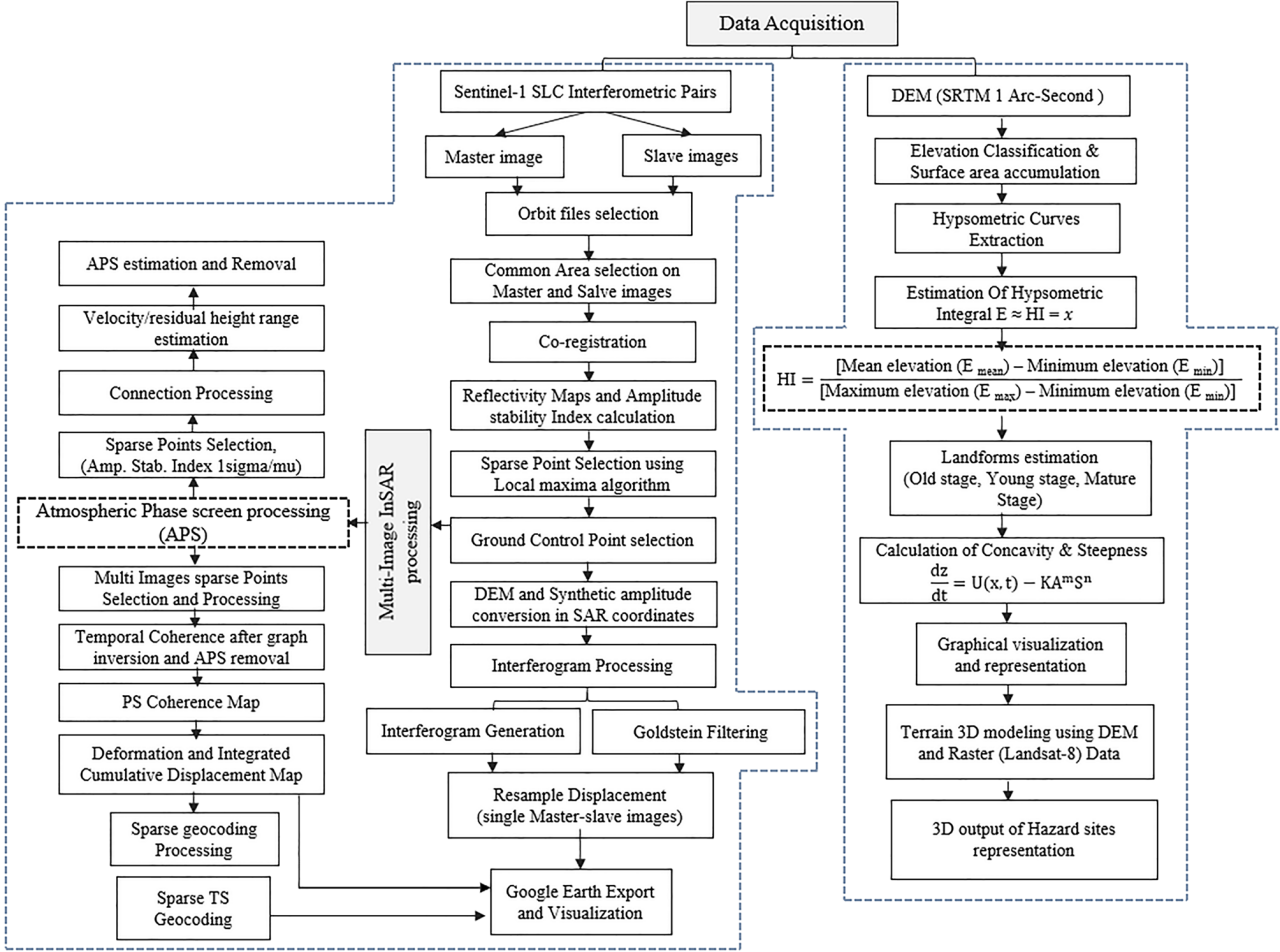
The Flowchart shows the overall processing steps which are applied for both PS-InSAR and hypsometry integral (HI) ANALYSIS used in this study.

The processing chain consists of Sentinel-1 and DEM data processing. Sentinel-1 interferometric pairs have the following components like the pre-processing sentinel-1 raw data; baseline estimation; co-registration; SPS (Sparse point Selection) using local maxima algorithm; Interferogram (single-multi) processing; Multi-Image InSAR processing; APS (atmospheric Phase screen processing); Sparse Point processing, coherence based cumulative displacement analysis and Time-series based deformation analysis. In a second chain, SRTM (1 arc-second, 30 m resolution) is used for the elevation classification based on elevation profiles and calculated hypsometric curves for each affected area. The elevation classification and area accumulation are used for the linear regression and the hypsometry integral $\mathrm{E}\approx \mathrm{HI}$ values extractions. The last landform type was estimated through HI values and multispectral 3D seismic topographic site indicated using the multispectral Landsat 8 data. An overview of the carried-out processing is shown in Fig. 2.

**Figure 3:**
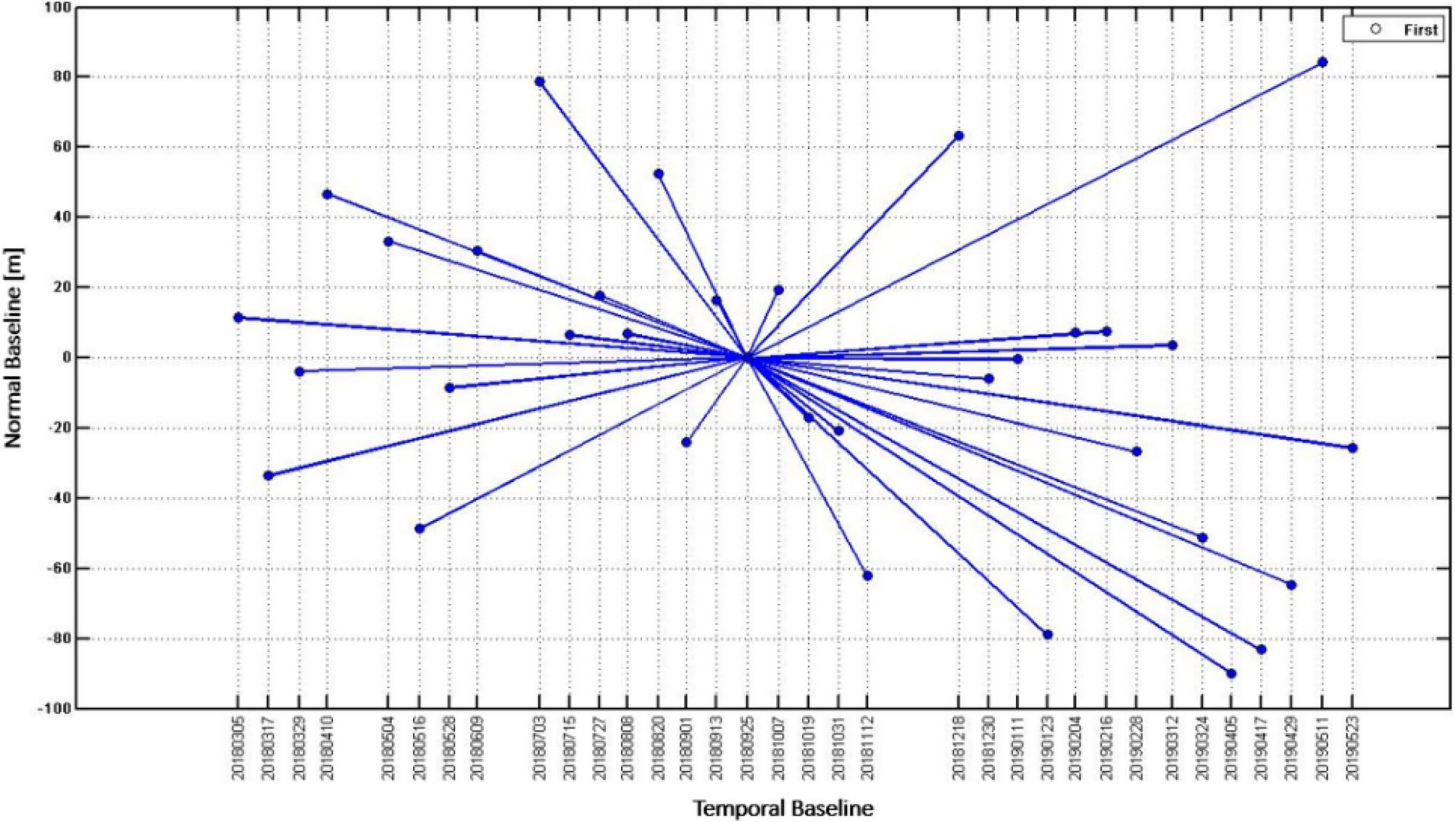
Graph drawing showing the temporal/normal baseline information of datasets from 5 March 2018 to 23 May 2019 of (Pre, co, and post) seismic event, blue lines represent the Interferogram of SAR acquisitions in blue dotes, Normal baseline on Y-axis and temporal baseline X-axis.

As the perpendicular baseline of the acquired images exhibits the coherence difference between the images. The smaller the existing perpendicular baseline between the master and slave images maintain the better overlap and demonstrate the effects of coherence among the images. The coherence has indirect proportionality using the perpendicular baseline, as with the decrease of perpendicular baseline, the coherence increases^26^. It is also recommended that to use pairs of acquired images with less than 600 m baseline values for attaining well coherence and overlaps between the salve and master datasets^27^. Hence, this study used the pair of sentinel-1 SAR images using normal minimum baselines ranging between 82.1 and − 90 m while ensuring better overlap. The pairs of sentinel-1 images below and above this range were excluded from the resultant SAR data processing. The graph exhibits the temporal/normal baseline information of datasets from 5 March 2018 to 23 May 2019 of (Pre, co, and post) seismic event of the study area as shown in Fig. 3.

After the baseline calculation, the Co-registration process was performed. Co-registration in sentinel-1 data processing was carried out by identifying the consistent pixels in both master and slave datasets. As in interferometric processing coherence is used for estimation^28^. Moreover, to attain every single imagery for the time series analysis, the co-registration process ensures that the same pixel resolution and grid gone through this process^29^. Afterward, the resultant interferograms showed the difference in phase between both co-registered master and slave images. The difference in variance in the form of colour fringes pattern on the interferogram are visible. Moreover, the idea about the generation of the interferogram is adopted from^30^ Eq. (1):

1 $${\mathrm{int}}_{(\mathrm{i}.\mathrm{k})} = {\mathrm{img}}_{(\mathrm{j})}\mathrm{ X }{\mathrm{img}}_{(\mathrm{k})}^{\mathbf{*}}.$$

In Eq. (1), ${\mathrm{int}}_{(\mathrm{i}.\mathrm{k})}$ indicating the interferogram taken between two images $\left(\mathrm{i}.\mathrm{k}\right)$, ${\mathrm{img}}_{(\mathrm{j})}$ a notation indicating the complexity of master and ${\mathrm{img}}_{(\mathrm{k})}^{*}$ is for the complex conjugate of the slave imagery.

With the increases in terrain value, the phase value stays wrapped between the range from 0 and 2π radians, and cyclic get repeated. This phase information of the interferogram has already been unwrapped. It then regulates the absolute phase value taken from the relative phase value of the interferogram because of adding and multiplying integer of 2π to the edges color fringe pattern. The information about the phase unwrapping phase has given by R. Burgmann^31^ in Eq. (2):

2 $${\mathrm{\varnothing }}_{\mathrm{unw}}={\mathrm{\varnothing }}_{\mathrm{wrapped}}+2\mathrm{n\pi },$$

where, ${\mathrm{\varnothing }}_{\mathrm{unw}}$ is the unwrapped phase, ${\mathrm{\varnothing }}_{\mathrm{wrapped}}$ is the phase wrapped, and the value of n is taking any positive integer. Whereas the process of interferogram phase unwrapping is used to filter and remove the effect of noise contributions in a resolution cell that contributed from the different sources. The Goldstein filtering technique is used for this processing. Goldstein’s filtering method is used to remove the noise present in the resultant products.

#### PSI (permanent scatter interferometry) approach

**Figure 4:**
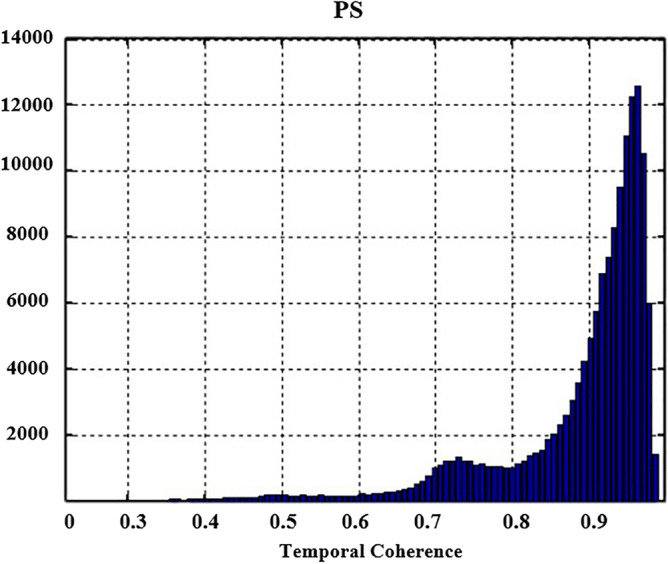
Temporal coherence of PS (permanent scatter).

Previously, Interferometric Synthetic Aperture Radar-PSI technique has been utilized to show deformation movement in the vulnerable zones using models, which in results showed the high Line of Sight (LOS) deformation velocity and land subsidence prediction at high susceptible regions^32,33^. In our study, for the selection of PS points, the amplitude stability index thresholding method is used. A threshold of 0.75 was chosen for permanent scatters as these scatters remain coherent throughout the period. While these permanent scatters (PS) have constant amplitude values in SAR datasets acquired from March 2018 to May 2019. The simplified Permanent Scatter approach fully utilized the coherence values from the temporal baseline of the images mainly in mountainous areas and gives the best results in high coherence areas with the rapid decrease in coherence value with time^34^. The temporal coherence of Permanent scatter (PS) is shown in Fig. 4.

The main procedure consists of the following steps:

- 2D sparse LS phase unwrapping using interferograms of the co-seismic event with and without Goldstein 15 × 15 window size.
- ScanSAR interferometric-based analysis for the identification of (pre, co, and post) seismic events with maximum vertical displacement values. The processing chain of PSI consists of multiple segments; Removal and estimation of the atmospheric component phase can be done using a set of Spatio-temporal filtering techniques^35–39^, Renovating the phases into displacements values and generating the time series-based deformation maps using the atmosphere attenuation free interferograms through the APS removal method.
- Afterward, the identification of the PSC (Permanent scatter candidates) and removing the corresponding APS (atmospheric phase screen) attenuation, the estimation of correlation between the PSC, and the map generation of the resultant PS coherence-based deformation. The last PSI processing approach generated the sparse time series deformation maps with their accumulated displacement values.

#### Hypsometric integral analysis

**Figure 5:**
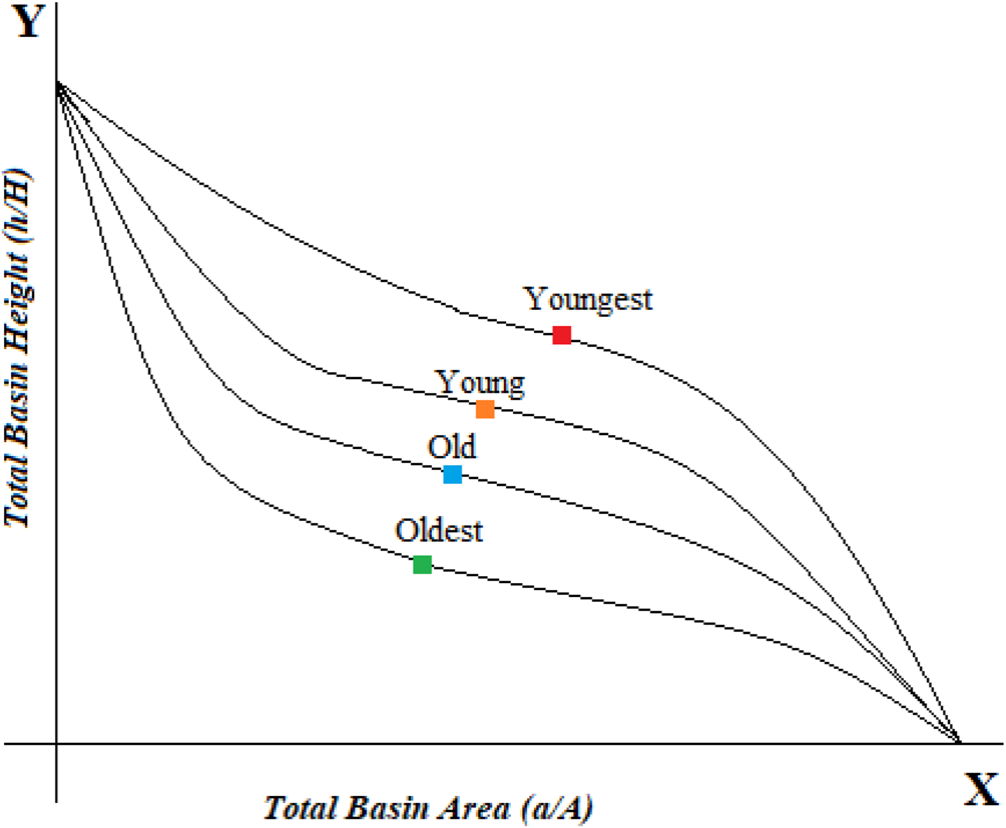
Interpretation of hypsometric curves showing youngest (red), young (orange), old (blue), and oldest (green) basins.

The hypsometric curve refers to the division of heights over an area of land. To estimate the progressive status of landforms vulnerability which is strongly associated with the extent of uplifting and erosion that has taken place in a given region^36^. The method of Hypsometric curves interconnected and correlated to tectonic evolution and geomorphic status of the basins in terms of their progressions and placements^37^. The most valuable feature of the hypsometric curve is the geomorphic status of the basins, and its diverse dimensions can be related to each other with resembled relative area and height ratios which are the impulsive functions of the total height and total area. However, the hypsometric integral curve values are fully independent of inconsistencies invariance, dimensions, and deviations^37^. The N. Strahler has classified the types of landforms using hypsometric integral values with the typical basin dissection stages. For this purpose, the hypsometric percentage method has been used to plot the hypsometric curves. It determines the relationship involving various variables plotted against one another. In curves, the *y*-axis shows the relative to total elevation (h/H) values, and the x-axis represents the relative to total area (a/A) information, as shown in Fig. 5. Additionally, the relative elevation is defined as the height of a given contour (h) ratio from the even elevation to the basin's maximum elevation (H). The relative area values are taken as a proportion value of the upper contour zone (a) to the total surface outlet area of the basin (A). The resultant correlation has a range from zero to one where the lowest value of zero is at the lowest point of the drainage basin (h/H = 0), and the highest value of 1 is at the uppermost point of the drainage basin (h/H = 1)^38^. The ordinate represents the relative to total elevation (h/H) data ratio. The abscissa represents the total to relative area (a/A) ratio as shown in Fig. 5 where concave up and convex down curves exhibit youngest, young, old, and oldest basins lithology type.

#### Approximation of HI (hypsometric integral)

This approximation of integration is adopted from the hypsometric curve that gives the hypsometric integral (HI) values equal to the ratio of elevation and relief (E) as suggested by Pike and Richard^39^.

Mathematically, it is defined as in the Eq. (3):

3 $$\mathrm{E}\approx \mathrm{HI}=\frac{{\mathrm{E}}_{\mathrm{mean}}-{\mathrm{E}}_{\mathrm{min}}}{{\mathrm{E}}_{\mathrm{max}}+{\mathrm{E}}_{\mathrm{min}}}.$$

In Eq. (3), E refers to elevation-relief ratio, HI = hypsometric integral value, E_mean_ defines the mean elevation; E_min_ is for minimum elevation, and E_max_ taking as maximum elevation. The HI value is orderly precise by the basin geometry and the area and relief ratio of the basin^40,41^ and inversely associated with the total value of the steepness relief of the basin, plus channel gradients. The hypsometric integral values quantify the phases of geologic lithology expansion and erosional status. The highest range of integral corresponds to the stage of youthfulness and less eroded landform areas. The lowest integral values demonstrate the landscape is towards its maturity and old stage of the basin. The integral value is taken as an indicator of percentage in the residue of the current paralleled volume and the original volume of the area basin and a complete cycle of erosion of the basin. This entire phase or cycle of basin erosion is assembled into three classes, each demonstrating the three distinct types of the basin stages to its geomorphic cycle. The first one is known as the Monadnock stage with the integral value (HI ≤ 0.35), where the basin is completely alleviated and stabilized. The second stage is the mature stage with the integral value of (0.35 ≤ HI ≤ 0.50). The third and last stage is the young stage which exists at (HI ≥ 0.50 or HI ≥ 0.60) integral range value, where the basin is exceedingly susceptible to the process of erosion^41^.

### Combined analysis of PS-InSAR and HI

The results obtained from the PS-SAR method were compared to the yield of measured geomorphic hypsometry integral curves values on the deflected streams networks. The combined SAR (remote sensing) and HI (geographical information system) results have provided evidence of the presence of active faults.

Moreover, the integral values in the study areas show differential erosional rates, and InSAR-derived results show the deformation trend in these regions with their annual velocity rates. The hypsometry technique and PS-InSAR results will help to provide information about seismically active locations and deformation status in the future.

## Results

### PS-InSAR-derived results

**Figure 6:**
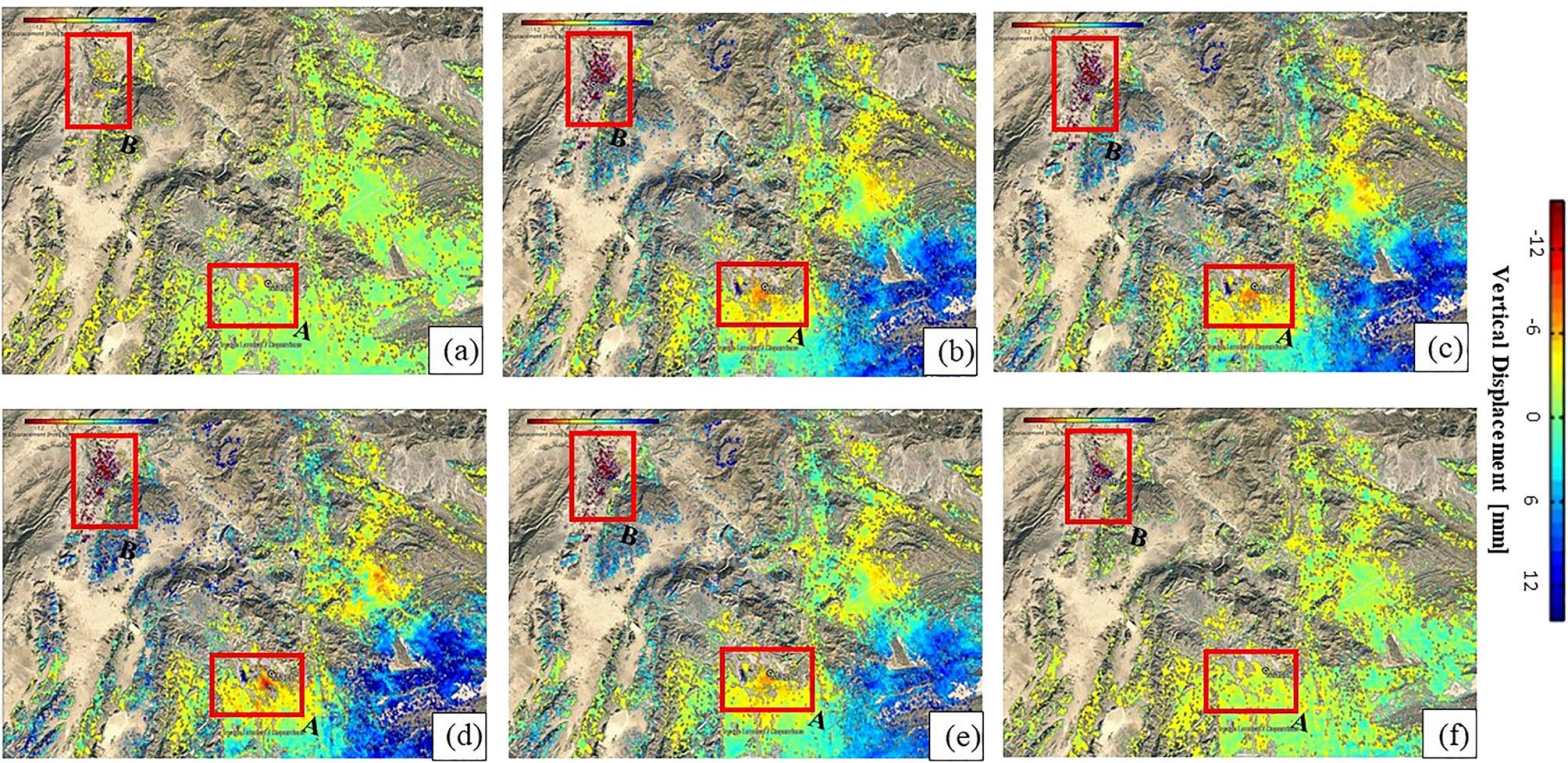
Shows the Pre, co and the post-seismic event Interferograms from March 2018 to May 2019 in Mach-A and Quetta-B city. Red rectangles are encompassed the affected region where (**a**) 05 March 2018 (Master) & 17 March 2018 (Slave) and (**b**) 05 March 2018 (Master) & 04 February 2019 (Slave) are pre-seismic interferograms, (**c**) 05 March 2018 (Master) & 15 March 2019 (Slave), (**d**) 05 March 2018 (Master) &19 March 2019 (Slave) are co-seismic interferograms and (**e**) 05 March 2018 (Master) & 04 May 2019 (Slave) and (**f**) 05 March 2018 & 23 May 2019 (Slave) are post-seismic interferograms. (© Basemap used from Google Earth platform).

PS-InSAR technique has been used for displacement analysis and given results were examined in a selected pair of sentinel-1 IW data taken for (pre, co, and post) seismic events from March 2018 to May 2019, as shown in Fig. 6. The study area from top to bottom along the vertical direction consists of sand and hard rock landform. The estimated results show that there is a constantly increasing trend in uplifting, which is observed just before the earthquake areas, as shown in red rectangles of Fig. 6. During the co-seismic event, the rate of uplifting gradually decreased and shifted temporarily to subsidence represented in red regions in red rectangular areas. Afterward, in a post-earthquake situation, areas seemed to recuperate the pre-earthquake uplifting trend in both Mach-A and Quetta-B city because of the strong reactional forces.

It is clear to note that in Fig. 6, there was an increase in the uplifting trend found before the earthquake (positive values), as shown in Fig. 6a. During the earthquake, it is clearly shown in co-seismic differential interferograms Fig. 6b–d that the rate of uplifting decreased, and in the meantime, the trend shifted towards subsidence (negative values) as shown in. Generally, there is a clear shift in the vertical displacement values between 12.0 and + 2.0 mm in along with the zero-reference line, and the topography in these areas mainly consists of thick stones. In the post-seismic interferograms Fig. 6e,f, it clearly shows that the displacement area has retained its original position, such as in pre-seismic events, because of strong reactional forces.

#### Time series analysis of surface deformation

**Figure 7:**
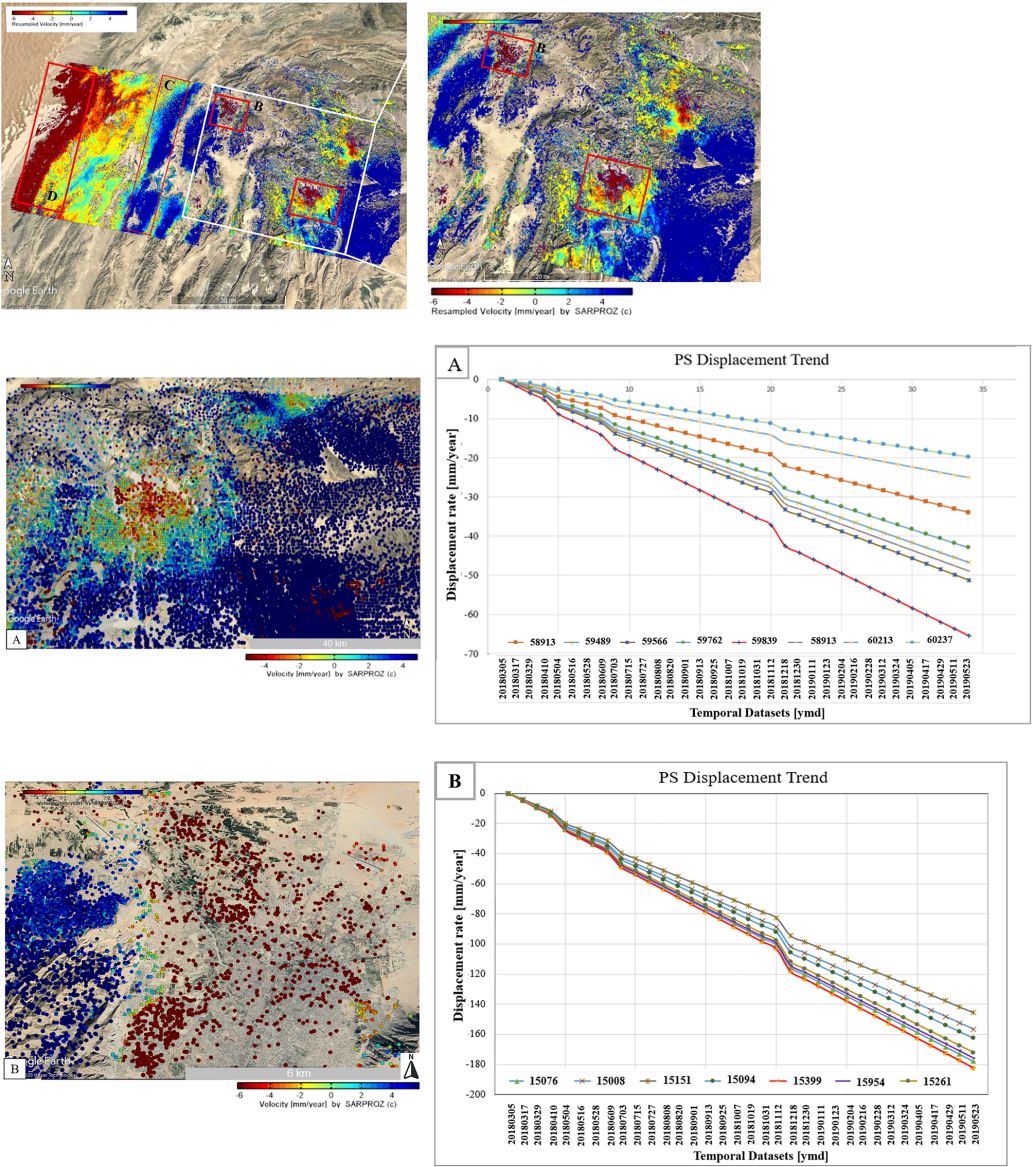
Shows the time series deformation maps along LOS direction using Sentinel-1 IW data along with Resampled velocity maps from March 2018 to May 2019 in descending mode. (**A**) Mach-A area, red rectangles are the area of earthquake Mw 5.0 Mach, 2019 along with PS displacement rate [mm/year] and 44 most protuberant coherent PS points spanning from March 2018 to April 2019. (**B**) Quetta-B area, red rectangles are showing the area of earthquake Mw 5.1 in 2019 along with PS displacement rate [mm/year] and 34 most protuberant coherent PS points spanning from March 2018 to May 2019 in descending mode. (© Basemap used from Google Earth platform).

Time series-based surface deformation trends measured over an area of Mach and Quetta city that has undergone earth activities are shown in Fig. 7. In this situation, the PSI simplified approach permits obtaining off the highly measured density points over the area of interest. The earthquake occurred on 16 March 2019 in the Mach and Quetta region; for this purpose, 34 pair images taken from March 2018 to May 2019 were selected to estimate pre-, co-, and post-seismic deformation and displacement analysis.

Figure 7 illustrates the linear deformation time series analysis over areas associated with earthquakes in Balochistan using full PSI approach. The LOS (line-of-sight) linear velocity information calculated from the PSI time series datasets for the indicated area that is characterized by earthquake activities using descending track orbits. As the deformation is demonstrated in the LOS direction, the positive velocity (color bar) represents the ground movement toward the satellite. The non-positive (negative) color bar (yellow to red colors) demonstrates the motion away from the satellite. Figure 7A shows the results of the 5.0 Mw earthquake around Mach city. There are around 44 most prominent/protuberant PSC’s points along the hill of slide spot at high altitudes (location of Mach city) and approximately 34 PSC’s prominent points taken within the boundary of Quetta city associated with 5.1Mw earthquake. The 44 PSCs in Mach city have shown a maximum value of cumulative displacement of − 65 mm during the study period of 22 months from March 2018 to May 2019, with an extreme decline in velocity rate up to − 4.0 mm/year along the line of sight (LOS). In Quetta city, 34 PSCs have shown a maximum cumulative displacement of − 97.7 mm during the earthquake with a total decline velocity of − 6.0 mm/year along LOS as shown in Fig. 7B. In the rest of the blue portion, an uplift with the rate of 4.5 mm/year has been dominated and most PSCs showing total cumulative displacement values from 13.6 to 17 mm/year. Some protuberant coherent PSC’s points with the Sigma Height, Velocity, Cumulative Displacement, Coherence, and Standard Deviation values observed in the city of Mach and Quetta obtained from the given datasets are shown in Tables 2 and 3. According the given results, a trend of increase in displacement (subsidence) shown with the passage of time from March 2018 to May 2019. PSCs of both areas are showing a linear trend of surface subsidence trend from March 2018 and subsidence rate is maximum in May 2019.

**Table 2:** Mach-City: few protuberant coherent PSC’s points with the sigma height, velocity, cumulative displacement, coherence, and standard deviation values observed in the city of Mach have been obtained from the given datasets.

PS points	Sigma height	Velocity	Sigma velocity	Cumulative displacement	Coherence	Standard deviation
58,913	7.43	− 27.96	1.86	− 33.99	0.92	2.08
59,453	7.36	− 37.37	1.92	− 45.43	0.94	1.73
59,489	7.23	− 38.4	1.88	− 46.68	0.95	1.59
59,566	7.07	− 42.1	1.84	− 51.17	0.9	2.22
59,689	7.82	− 56.46	1.95	− 57.85	0.96	1.35
59,762	7.79	− 35.31	1.88	− 42.92	0.94	1.6
59,839	8.41	− 53.84	1.96	− 65.45	0.94	1.72
59,859	7.91	− 40.22	1.94	− 48.89	0.94	1.72

**Table 3:** Quetta City: few protuberant coherent PSC’s points with the sigma height, velocity, cumulative displacement, coherence, and standard deviation values have been obtained from given datasets.

PS points	Sigma height	Velocity	Sigma velocity	Cumulative displacement	Coherence	Standard deviation
15,151	7.81	− 1562.36	− 119.75	− 145.57	0.67	34
15,008	10.17	− 1586.5	− 128.9	− 156.69	0.63	34
15,094	10.46	− 133.67	2.01	− 162.49	0.72	3.95
15,261	9.58	− 141.57	2.48	− 172.09	0.49	5.47
15,954	11.08	− 144.53	2.03	− 175.69	0.6	4.72
15,399	9.26	− 150.87	2.11	− 182.34	0.56	5.11
15,076	10.64	− 146.35	2.44	− 177.9	0.65	4.35

### Hypsometry integral values calculation for seismic susceptibility

**Figure 8:**
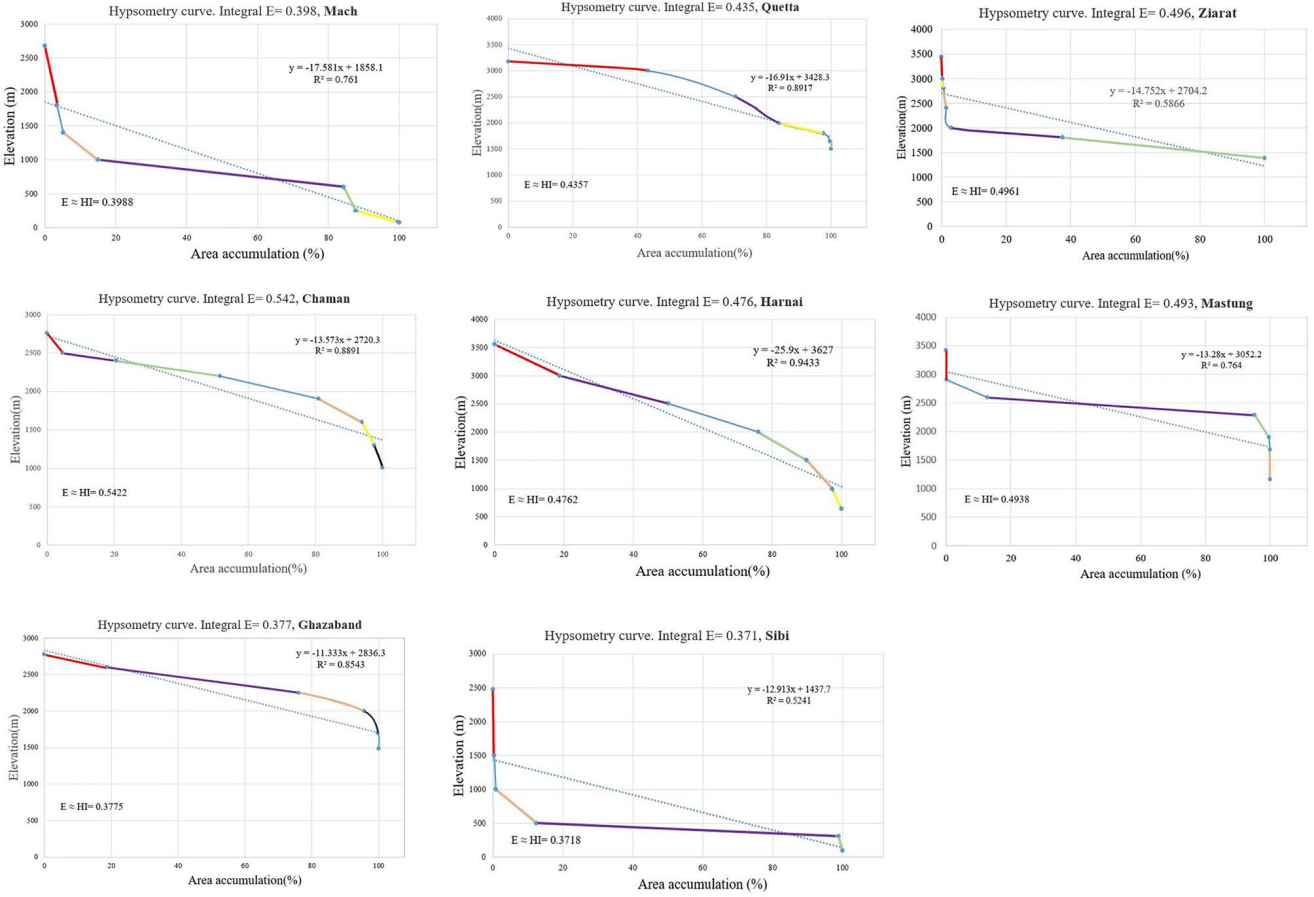
Hypsometric curves along with Integral values (E ≈ HI), Linear regression ($\mathrm{y}=\mathrm{\alpha }+\mathrm{\beta x}, {\mathrm{R}}^{2}$), each colored line represents a sharp change in channel slope (Knick Points).

There are approximately nine hypsometric curves extracted at the surrounded areas of Mw 5.0 earthquakes in Mach and Quetta city to evaluate the seismic landscape characteristics and status of landforms that are strongly associated with the extent of erosion and uplifting. The gentle and steepening of slopes throughout the basins escorted by a more rapid rate change in elevation to a change of horizontal cross-sectional area. The hypsometry curves of Mach, Sibi areas exhibit steep part to the elevation in the beginning and then relatively gentle slopes belts with the integral values of HI_Mach_ = 0.398 and HI_Sibi_ = 0.4. This shows that the basin is at equilibrium stage where the rate of differential erosional stages is equal to the neo-tectonic level which are expressed in Fig. 8.

Figure 8 demonstrate that the hypsometric curves in the rest of surrounded areas signifying the level of neotectonic activity are nearly dominating in Mastung and Ziarat’s city. The fault regions and the curves are convex-up shaped with the integral values of HI_Mastung_ = 0.49 ≈ 0.5 and high in the Chaman area with HI_Chaman_ = 0.54. As the Chaman fault and its bounded areas are the locus of many catastrophic earthquakes with inception strike-slip movement at 20–25 Ma along the western collision boundary of Indian and Eurasian plates. The average observed geologically constrained slip rate was about 24 to 35 mm/year, accounting for a total displacement of 460 ± 10 km along the Chaman fault area. The intermediate steeper part of the hypsometric curve of Quetta, Chaman, and Harnai areas corresponds with valley wall slopes in the midsection of the basin, and the steeper part at the very lowest part of the curves with its lower elevation point is the indication of the mouth area of the basin. The hypsometry curves of each relevant city basin indicate that the equilibrium rate is more dominant in the areas of Mach, Quetta, Ghazaband, Sibi, Harnai with the integral values of 0.35 ≤ HI ≤ 0.50. Moreover, the Chaman, Mastung, and Ziarat basin indicates the basin is near to susceptible to erosion and exists at a young stage. However, no basin in this region was at Monadnock stabilized stage condition with the integral value of HI ≤ 0.35.

#### Topographic elevation profiles EP

**Figure 9:**
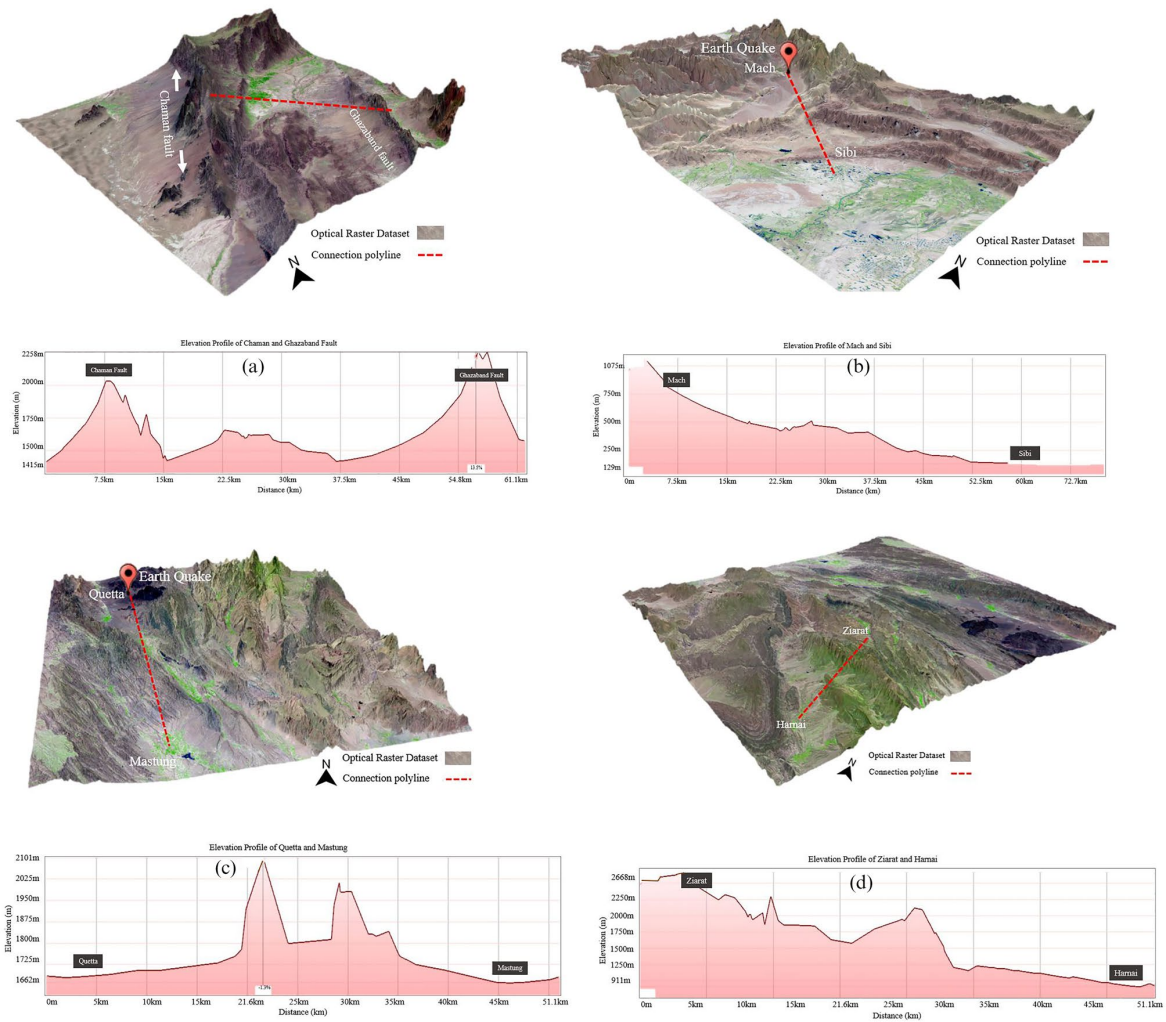
(**a**) Elevation profile (EP) of Chaman fault (2100 m) to Ghazaband fault (2258 m) with; (**b**) Mach-A (810 m) to Sibi (129 m); (**c**) Quetta-B (1665 m) to Mastung (1660 m); (**d**) Ziarat (2668 m) to Harnai (911 m). 3D seismic topographic sites were indicated using the multispectral Landsat 8 data (30 m) and SRTM DEM (30 m). (© Basemap used from Google Earth platform).

Elevation Profile was extracted using the Digital Elevation Model (DEM) and Landsat 8 data to identify the 3D topography visualization of landscape characteristics. At the same time, the elevation profile between Chaman and Ghazaband fault shows the steepening of slopes from 2100 to 2258 m and the mid-section accompanied by the total elevation from 1420 to 1650 m by a more rapid rate in the decrease of elevation as shown in Fig. 9a.

The elevation profile between Mach (810 m) and Sibi (129 m), at first thought, suppose that at steeper parts, the curve is towards Mach and ultimately coincides with the belts of relatively least gentle slopes curve of Sibi area with 130 m elevations as shown in Fig. 9b. Whereas in Fig. 9c, the elevation profile between Quetta (1665 m) to Mastung (1660 m) shows two big peaks in the middle of profile at 2101 m and second at 2020 m. Moreover, in Fig. 9d of Ziarat (2668 m) to Harnai (911 m), peak-valley trend could be seen from Ziarat-2668 to 2125 m. Afterwards, big dip comes in centre and slope becomes less steep from 1250 to 911 m until Harnai. In contrast, reports of major earthquakes in the surrounding cities, e.g., Quetta and Mach, showed widespread damage to cities and ground disruption. Therefore, this study implies the earthquake assessment and vulnerability analysis using geomorphological examination, Sentinel-1 based PS InSAR technique, HI, and hypsometric curves (HCs) were used to interpret the recent, youthful, and active surface deformation areas and their influence on the local topography of Balochistan.

### Combined PS-InSAR and hypsometry integral (HI)

The obtained results from the combined InSAR and Hypsometry Integral approach suggested that the area of Quetta (B) and Mach (A) in Fig. 7. It experienced the subsidence with a total velocity rate − 4 to − 6 mm/year with the mature Hypsometric Integral values where the rate of uplift is nearly equal to the rate of erosion as shown Fig. 8. This region will experience a modest seismic forthcoming rate in the future and be susceptible to both erosion/uplifting with a velocity rate of more than − 6.0 mm/year. Previously this region experienced a velocity rate defined as − 5.8 mm/year. This velocity is used to identify the impact of tectonic processes on subsidence and the pattern of earthquakes in the same region^42^. Secondly, the Chaman (D) and Ghazaband (C) faults areas result from the north–south striking left-lateral transform boundary zone, which makes this region more susceptible for future seismic activities with a velocity rate more likely than the existing ~ 6 ± 4 mm/year.

Moreover, the slip rate of 8 mm/year from 5 years of GPS observations near Chaman City and the observation GPS station in Kabul was measured. Previously it accounts the 75% of the Indian and Eurasia plates motion in 2012^43^. The InSAR observations from the topographic residuals and stratified tropospheric delay show that the Chaman Fault accommodates only ~ 8 mm/year of the relative motion. The deformation is partly accommodated by shallow fault creep at this segment of the Chaman Fault with a maximum surface creep rate of ~ 8 mm/year from the year 2004–2011^44^.

**Figure 10:**
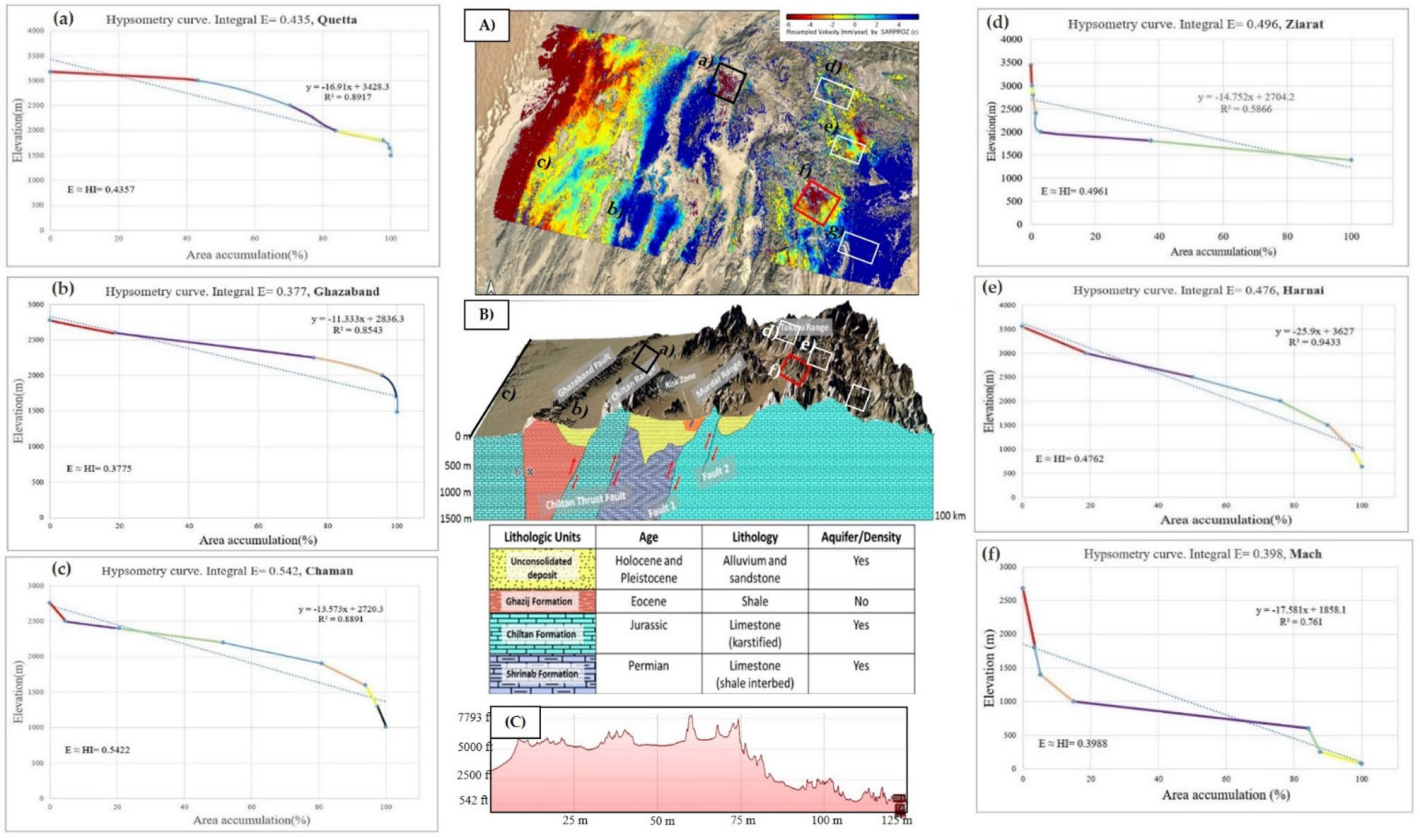
(**A**) Time series surface deformation map, (**B**) schematic cross-section of 3D topographic characteristics to explain Lithology in the surrounding areas and (**C**) elevation profile. (**a**) Integral profile of Quetta with the lithology units and elevation. (**b**) Ghazaband uplift value with convex up Integral value and limestone lithology. (**c**) Chaman fault with limestone lithology leading to higher convex up integral values with greater subsidence. (**d**) Ziarat with higher convex up integral values. (**e**) Harnai city towards subsidence. (**f**) Mach with lithology aquifer/density comparison to the subsidence. SRTM (1 arc-second, 30 m resolution) is utilized for Hypsometry Integral Analysis.

Using GIS techniques, the obtained InSAR deformation results were combined and compared to the measured geomorphic hypsometry integral curves values. Hypsometry Integral values of Quetta, Mach, Chaman fault refer towards the youthful, active tectonic regional area with an Integral values HI_Mach_ = 0.398 and HI_Quetta_ = 0.435 (0.35 ≤ HI ≤ 0.52); and HI ˃ 0.53 more youthful susceptible class region with S and convex-up shape. These results were previously noted in the Neogene to Quaternary sedimentary Valley, which provides a good technique for evaluating fault activity and related erosion processes^45^. These combined PS-InSAR and GIS results are evidence of the presence of active tectonics in this region and responsible for bringing out the surface deformational activities in these regions. The approach of active tectonic presence in the region using geomorphic HI values was previously distinctly identified by other researchers^46^. To compare Fig. 10A shows the time series surface deformation map. Figure 10B,C shows schematic cross-section of 3D topographic characteristics revised from a previous study^47^ and elevation profile to describe the Lithology in the neighbouring areas. Figure 10a shows the integral profile of Quetta with the value of E = 0.435. Whereas Fig. 10b explains the Ghazaband uplift value with convex up integral value of 0.377 and its limestone lithology which results in the most vulnerable site. Figure 10c Chaman fault with limestone lithology leading to higher convex up integral value of 0.542 with greater subsidence. Figure 10d explains the Ziarat region with higher convex up graph with integral value of 0.496. Moreover, in Fig. 10e Harnai city have integral value of 0.476 and its graph shows the site is towards subsidence and in Fig. 10f Mach having integral value of 0.398 alike to Ghazaband showing its active seismic properties. The rate along with Ghazaband fault Fig. 10c can be unprecedented towards uplifting with a total velocity more than the existing 5–6 mm/year. It has been proven previously by over a wider zone of Ghazaband Fault, which provided a total deformation rate ~ 16 mm/year from 2004 to 2011. Since 1870, after the four major earthquakes from the last 40 years long global centroid moment tensor (CMT) catalog: M_w_ 6.1; M_w_ 5.3 in 1990; M_w_ 5.6 in 1993; M_w_ 5.5 in 2007 makes the Ghazaband Fault one of the most hazardous faults in the plate boundary of Balochistan^44^. In comparison to Ghazaband, Mach have approximately same integral values as mentioned in Fig. 10f which clearly shows that region is as active as Ghazaband.

In this study, we have also proven that using the Hypsometric technique with the integral value for evaluating future seismic fault activity with the value of 0.377 towards subsidence. These hypsometry curves of Mach, Ziarat and Sibi areas are seen towards steep part to the elevation in the beginning and then relatively towards gentle slopes as shown in Fig. 8. In addition, the integral values in the corresponding regions of HI_Mach_ = 0.398 and HI_Sibi_ = 0.4 shows the rate of differential erosional rate is equal to the neotectonic level, and the deformation trend in these regions can exceed from the current rate up to ± 2.1 to ± 3.5 mm annual velocity rate in future. This hypsometry technique^2^, along with InSAR data, provided future seismic activity sites with deformation status and exposed that the integral values calculated by elevation–relief ratio method was accurate, less weight, and easy to estimate using GIS environment.

### Evaluation of results

**Figure 11:**
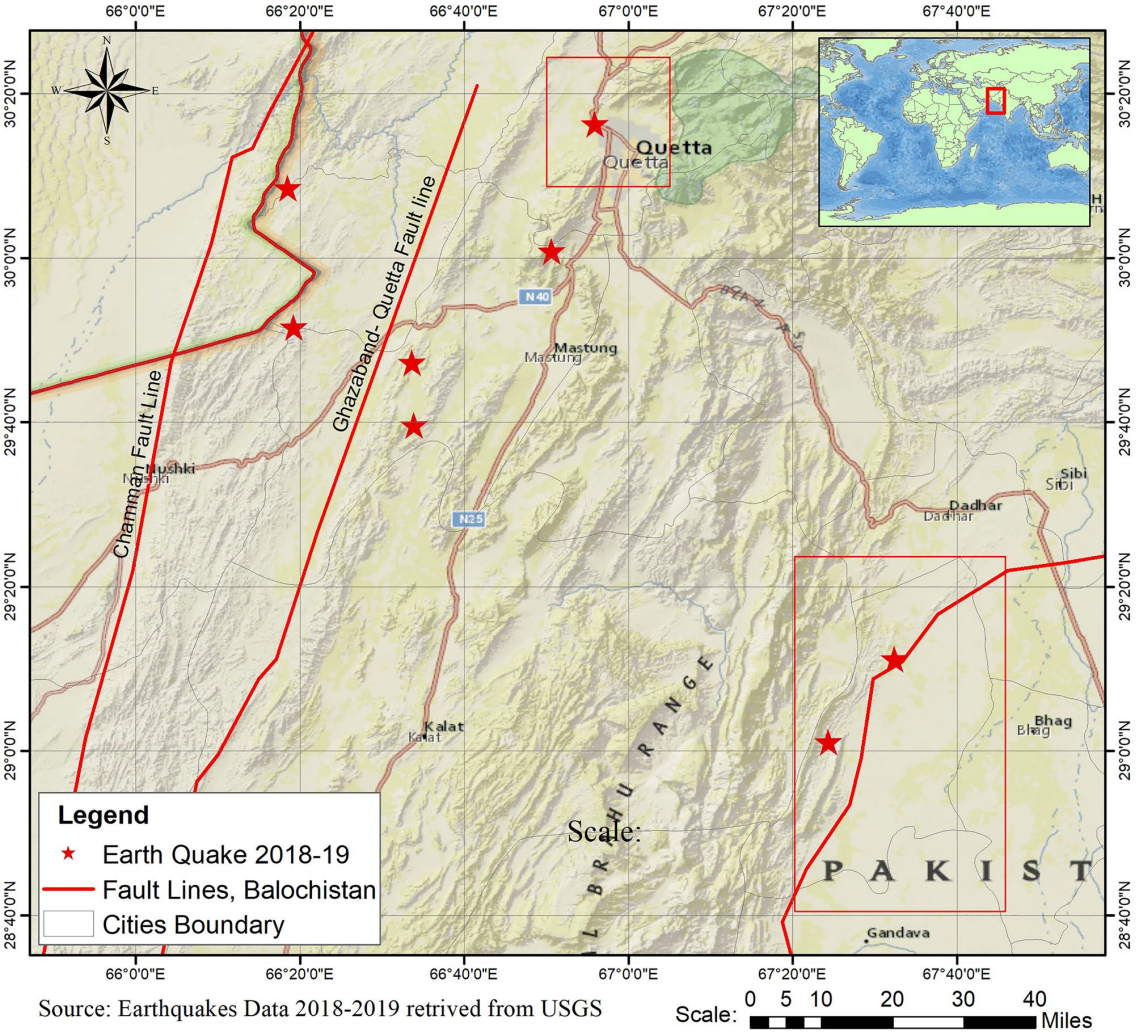
Shows the number of earthquakes stokes nearby Quetta and Mach areas in the years 2018 and 2019. Affected regions are indicated by a red star and red rectangles indicating the study areas; data retrieved from USGS.

The selection of study areas in Balochistan, Pakistan, was done from the reports of numerous international organizations that have been in process over a legitimately long time as shown in Fig. 11. Moreover, the GPS rates of the surface deformation were also retrieved in the Quetta Valley by the University of Balochistan, the Balochistan University IT Department (QTIT), the Balochistan University Geology Department (QTAG) with the data acquired using the four GPS monitoring sites in the valley. All the stations in the valley were not continuously operating and were recorded at an interval of two months or greater. Therefore, the ground-based GPS rates showed the deformation at around − 7.7 mm/year to identify the subsidence in the valley, which is closer to the displacement rate of − 6.0 mm/year, calculated in our study. However, the previous studies could identify the deformation up to − 5.1 mm/year rate. For better and more accurate results, we require more working stations data.

The earthquakes data used in this research paper was retrieved from the United States Geological Survey (USGS PDE), and Pakistan Meteorological Department (PMD) earthquake data catalogues as shown in Fig. 11. For identifying the total assessment in the affected earthquake region, persistent scatter interferometry (PSI) based approach has been used and inspected the total surface displacement before and after the events with help of Sentinel-1 time-series data.

The hypsometry curve technique is used to evaluate the area of seismic susceptibility sites. It is a handy tool for the identification of the most vulnerable sites. In the end, this combined approach allows us to identify the seismic areas related to young and old active tectonics sites based on the deflected streams data through google earth imagery.

**Figure 12:**
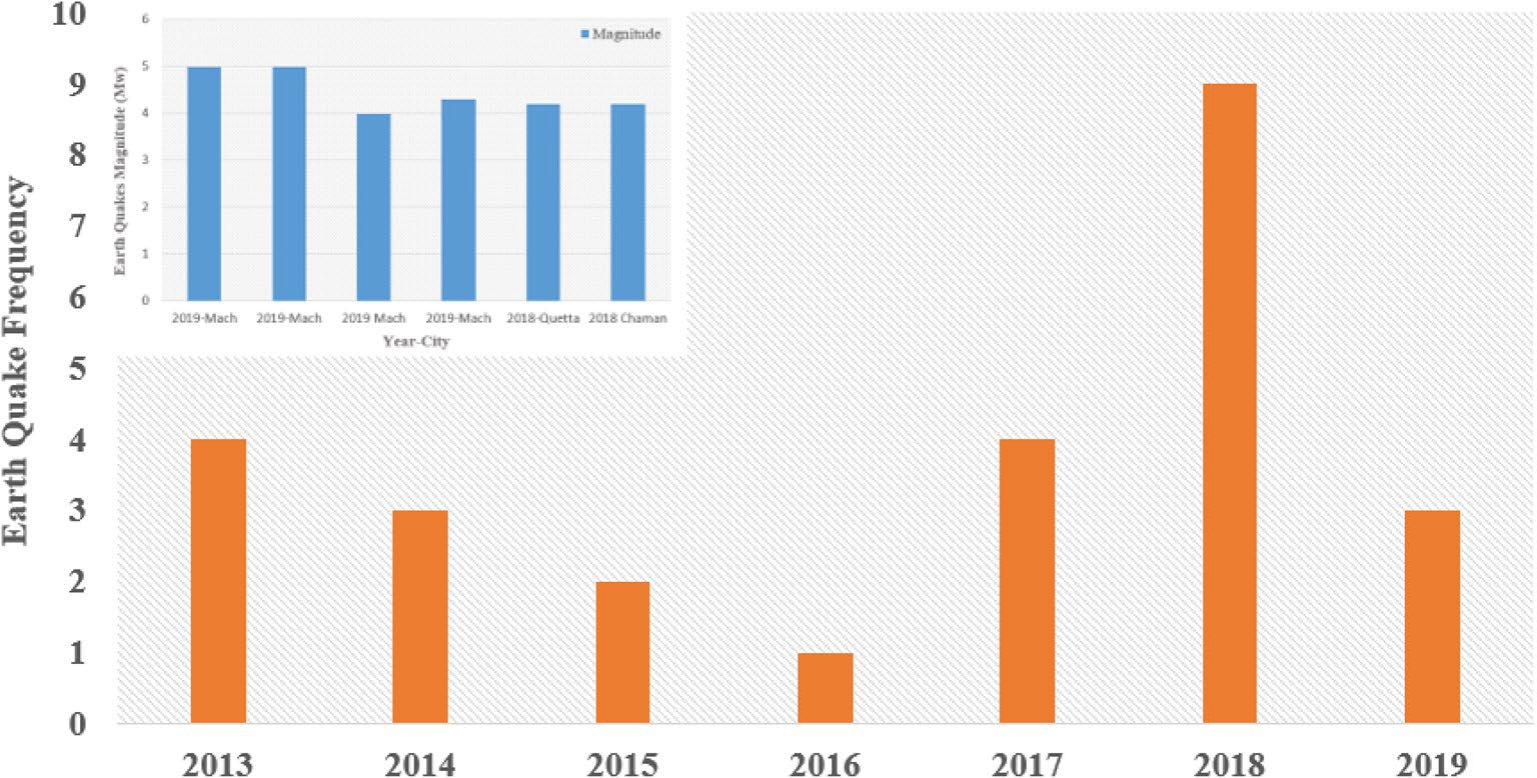
Earthquake frequency distribution in Quetta, Mach, and Chaman fault region since 2013 to 2019.

**Figure 13:**
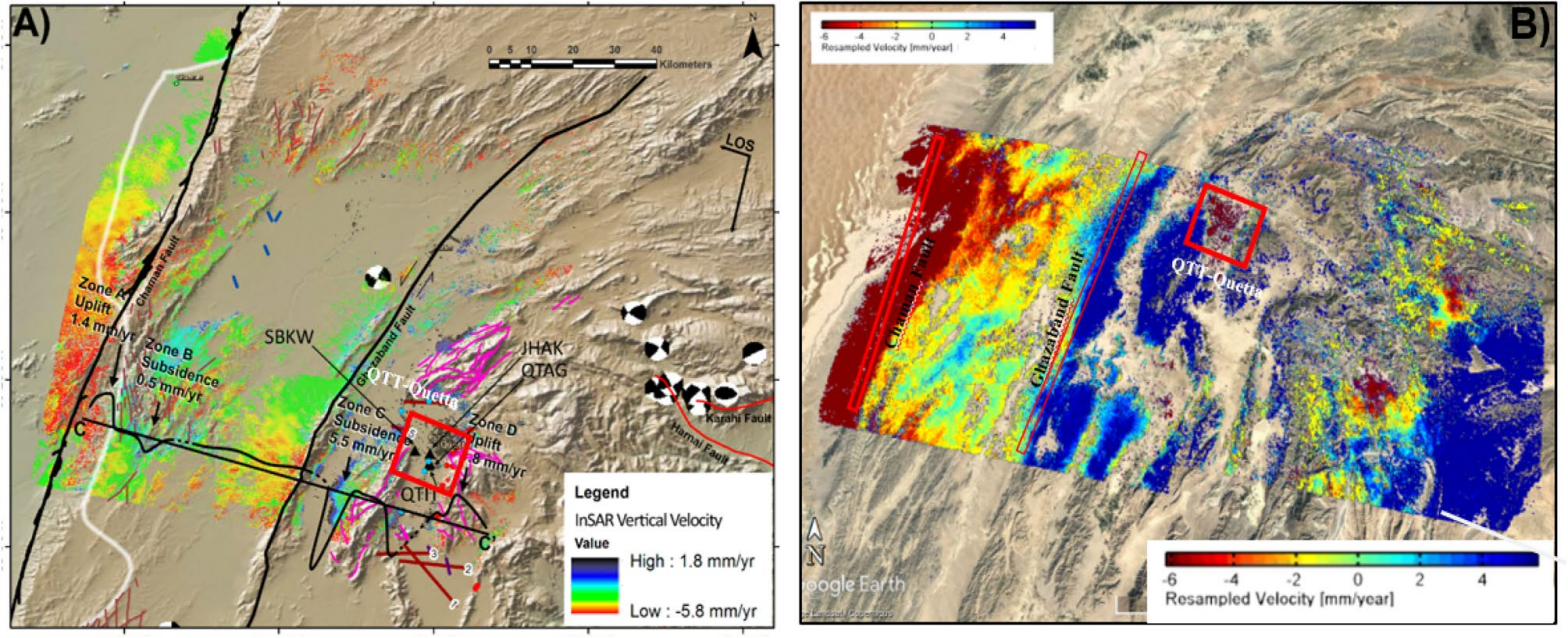
(**A**) InSAR-based results show the surface deformations ranging from − 5.8 mm/year were generated using forty descending images from 2003 to 2010. A dotted line marks the velocity profile ‘CC’ low coherence area. GPS rate from 2006 to 2016 in a vertical direction around − 7.7 mm/year identify the subsidence^42^. (**B**) Time series surface deformation along the LOS was estimated using 34 descending images in the affected earthquake Mw 5.0 area with a − 6.0 mm/year displacement rate. (© Basemap used from Google Earth platform).

The Chaman Fault (or fault group) is shown by the red color line boundary in Fig. 11, which stretches towards sub-N-S until it meets another fault named Herat in the north. Chaman Fault is a left-lateral strike-slip that has not unconfined a high earthquake activity that took place around this region in the recent past. The researcher speculates whether a substantial amount of accumulated stress has prepared this region for many more major earthquakes determined by the Ambraseys and Bilham^48^. Moreover, the Ghazaband fault area is also shown by the red line boundary along the city of Quetta Structure modelled as a single fault that runs parallel to the south of Chaman Fault but curves in the north and links with the Kalat–Chinjin Structure to the Suleiman lobe. The earthquake-influenced cities of Mach and Quetta Structure have also been indicated in the map with geological/fault structures. Surface manifestations strongly recognize these areas; the large magnitude of past earthquakes appear to align along with this structure. It may be responsible for the Quetta–Mach earthquake of magnitude 5.0. The evidence from the assessment and analysis in the recent research led to several large earthquakes that have rattled the area in the past and contributory epochs, as shown in Fig. 12. The major cause of these devastating earthquakes lies in the tectonic and fault systems (Chaman fault; Ghazaband fault) surrounding the region. The earthquake assessment and vulnerability sites were carried out in area sources, and in terms of fault system sources^49^. The obtained results through hypsometry curves integral values can be used for the identification of seismic sites and can ascertain the engineer’s future urban safe planning sites. The verification of PS InSAR analysis, all the results were firstly compared with the ones obtained from the previously conducted studies to estimate the surface deformation in the Quetta and Mach region as shown in Fig. 13.

#### Comparison of surface displacement results in the Quetta region: previous and recent studies

The previously conducted studies to estimate the surface deformation in the Quetta region have shown the vertical displacement velocity from the year 2006 to 2011. The displacement was observed at about − 5.8 mm/year as shown in Fig. 13A to identify the impact of tectonic processes and the pattern of earthquakes in this region^42^. The previous researcher also used the small baseline technique by combining differential interferograms to calculate surface deformation velocities by phase profiles after reducing atmospheric and orbital errors^27^. On the same descending path, the deformation trend with high coherent PS points spanning from 2018 to 2019 can be seen with a total vertical displacement velocity value − 6.0 mm/year subsidence on the right side of Fig. 13B. Positive values can be considered as a shift eastward or uplift. This movement is ascribed mainly to uplift for the following reasons: (1) Tectonics of the region is endangered to thrust and north–south strike-slip faults and overriding valleys. We could not resolve the north–south strike-slip Chaman Fault movement because the strike-slip movement is perpendicular to the Line of Sight (LOS) direction. (2) The GPS observations obtained from the stations are deployed in previous studies in Quetta Valley during the year of 2006–2016 that exhibit that observed deformation is mainly towards the direction of subsidence^42^, which is also proven in the recent InSAR data processing (2018–2019) during the deformation analysis of Mach-Quetta earthquakes.

**Figure 14:**
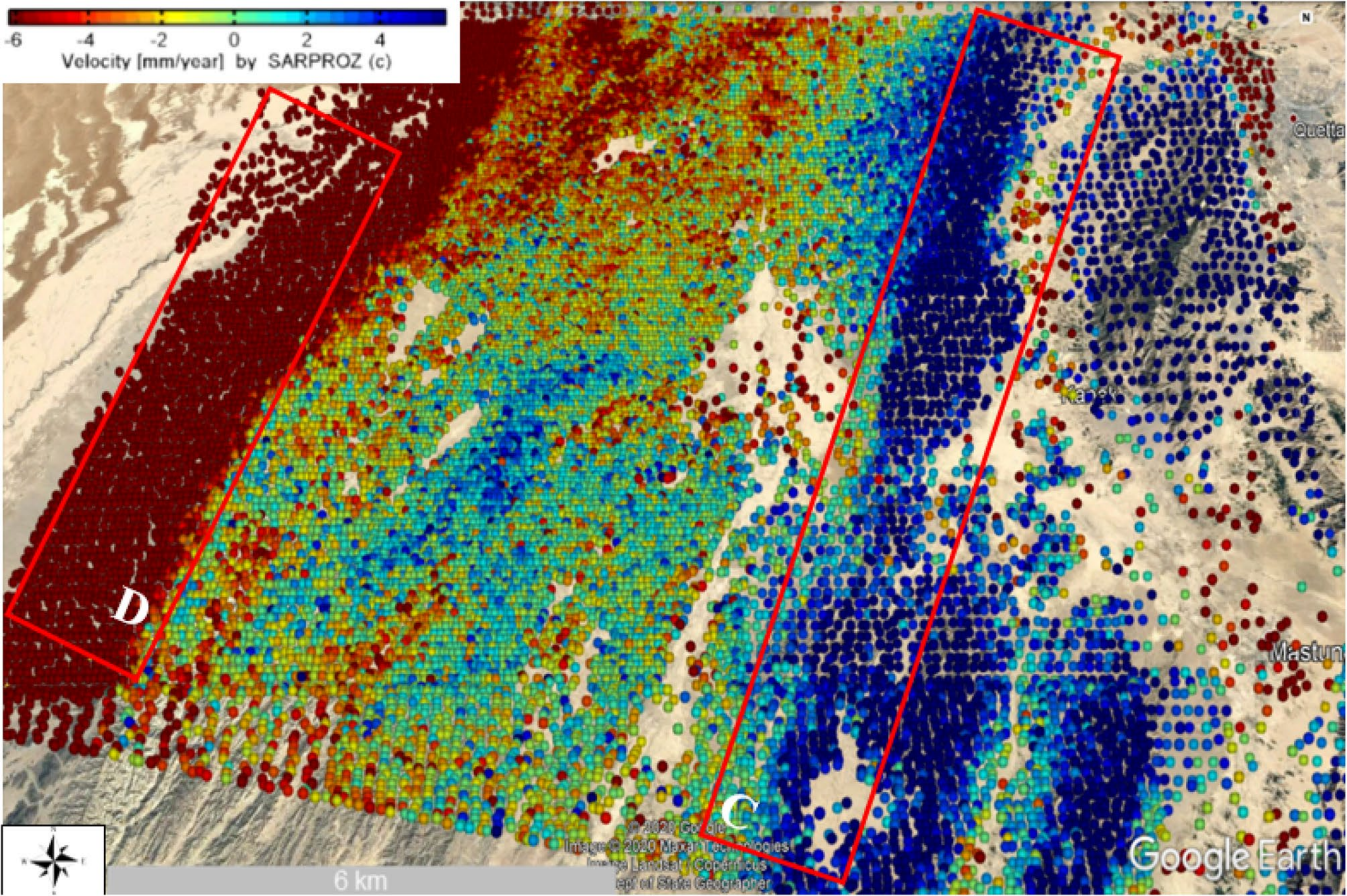
Time series surface deformation results of sentinel-1 datasets from 5 March 2018 to 23 May 2019 of (Pre, co, and post) seismic event along the LOS direction around Ghazaband fault (C) and Chaman fault (D). (© Basemap used from Google Earth platform).

**Figure 15:**
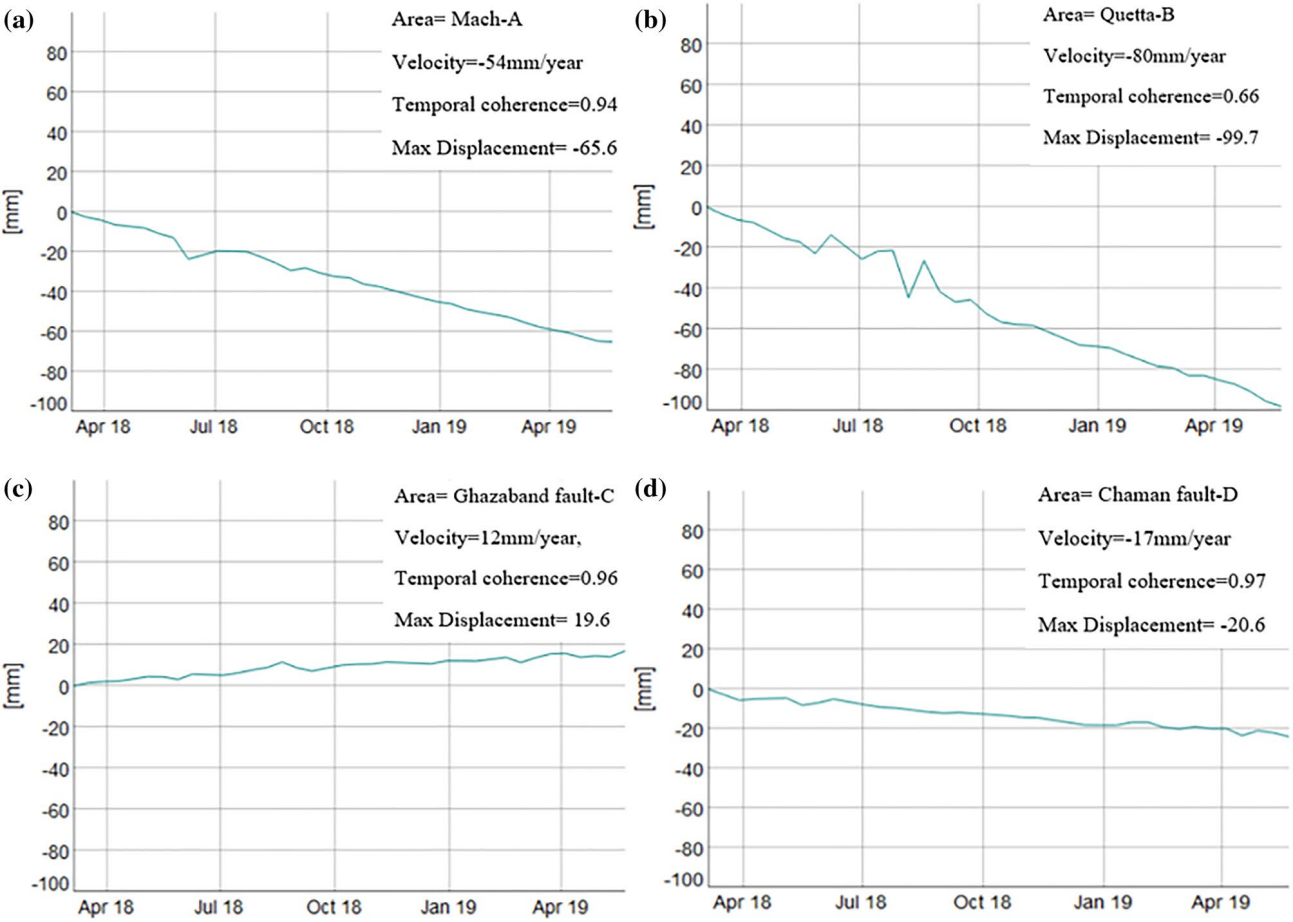
PS-InSAR time series displacement analysis (**a**) Mach-A Mw 5.0 earthquake, (**b**) Quetta-B Mw 5.0 earthquake, (**c**) Ghazaband Fault-C, (**d**) Chaman Fault-D.

This relative leading uplifting could be due to pressure increase in the Ghazaband fault along with the Quetta city and responsible for earthquake strike there as shown in Fig. 14. However, locations of uplifting and subsidence during the pre-seismic period were not stable in the study areas. After assembling the earthquake’s catalogue and time-series deformation analysis were performed, it has estimated the primary uplift/subsistence of earthquakes Mw 5.0 in Mach and Quetta. Mach and Quetta earthquakes have epicentres along with the Chaman and Ghazaband fault, respectively. Temporal variations in vertical displacement in Mach and Quetta cities may cause by one of the significant surrounded faults, Chaman and Ghazaband. Figure 15 shows the graphical representation of PS-InSAR time-series displacement analysis of Mach, Quetta, Ghazaband and Chaman in Fig. 15a–d respectively with its velocity, temporal coherence and max displacement values. In their comparisons, only Ghazaband fault gives the positive values for velocity and max displacement which explains the instability and more active seismic site with respect to others.

## Discussion

This section analysed and summarized the comparison results obtained through combined Persistent Scatter Interferometry (PSI) technique and Hypsometry Integral (HI) analysis in various regions of Pakistan. Most studies focused on analyzing earthquake-induced deformation using sentinel-1 based RADAR interferometry approaches^50–52^ that is why we used Mach and Quetta earthquakes for our study. This study is an attempt to evaluate and compare seismic vulnerability through combined PSI and HI techniques. For this purpose, openly accessed data was used, and the multi-temporal PS-InSAR method was applied for the displacement analysis^51^. The high temporal resolution enabled us to acquire radar images before and after the earthquake event. The data was processed and monitored with corresponding hypsometry integral values to identify the future tendency of seismic sites^53,54^. The processing of Sentinel-1(open access) satellite data is done using the open-source software SARPROZ provided by ESA and processing tool by Periz using MATLAB programming. Moreover, the Geographic Information Systems (GIS) techniques employed to process digital elevation model (DEM) data to extract the valuable mapping representation of all the different parameters and collected data, enabling the spatial representation of the various geomorphic findings^5^.

**Figure 16:**
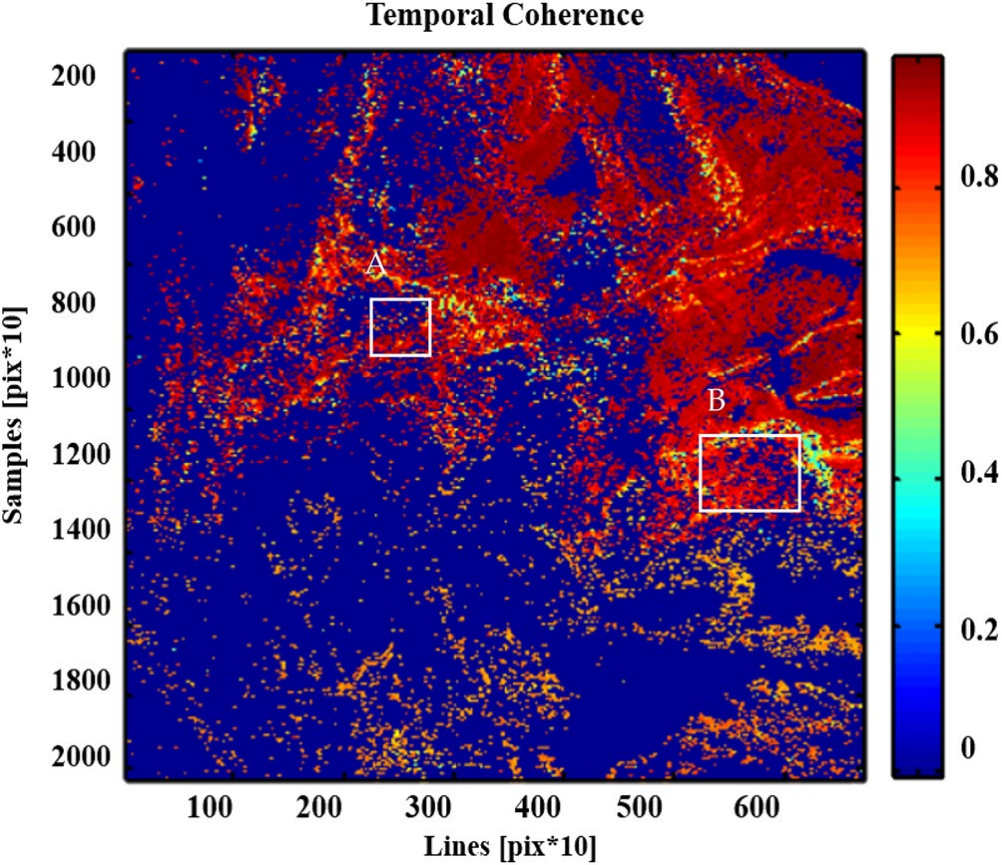
Variation in the temporal coherence of Mach-A and Quetta-B region indicated by white rectangles.

The result obtained from time-series deformation analysis indicates that the Mach-A city has observed the deformation as uplift of 7.56 mm/year and subsidence of − 53.84 mm/year. Quetta-B city experienced the deformation from − 150 mm/year to − 27.96 mm/year, as shown in Fig. 16. Additionally, the simplified approach is used in these affected areas based on the most protuberant coherent PSC’s points. A similar situation was observed during the Pasni earthquake, and the total surface deformation was estimated using RADAR interferometry.

**Figure 17:**
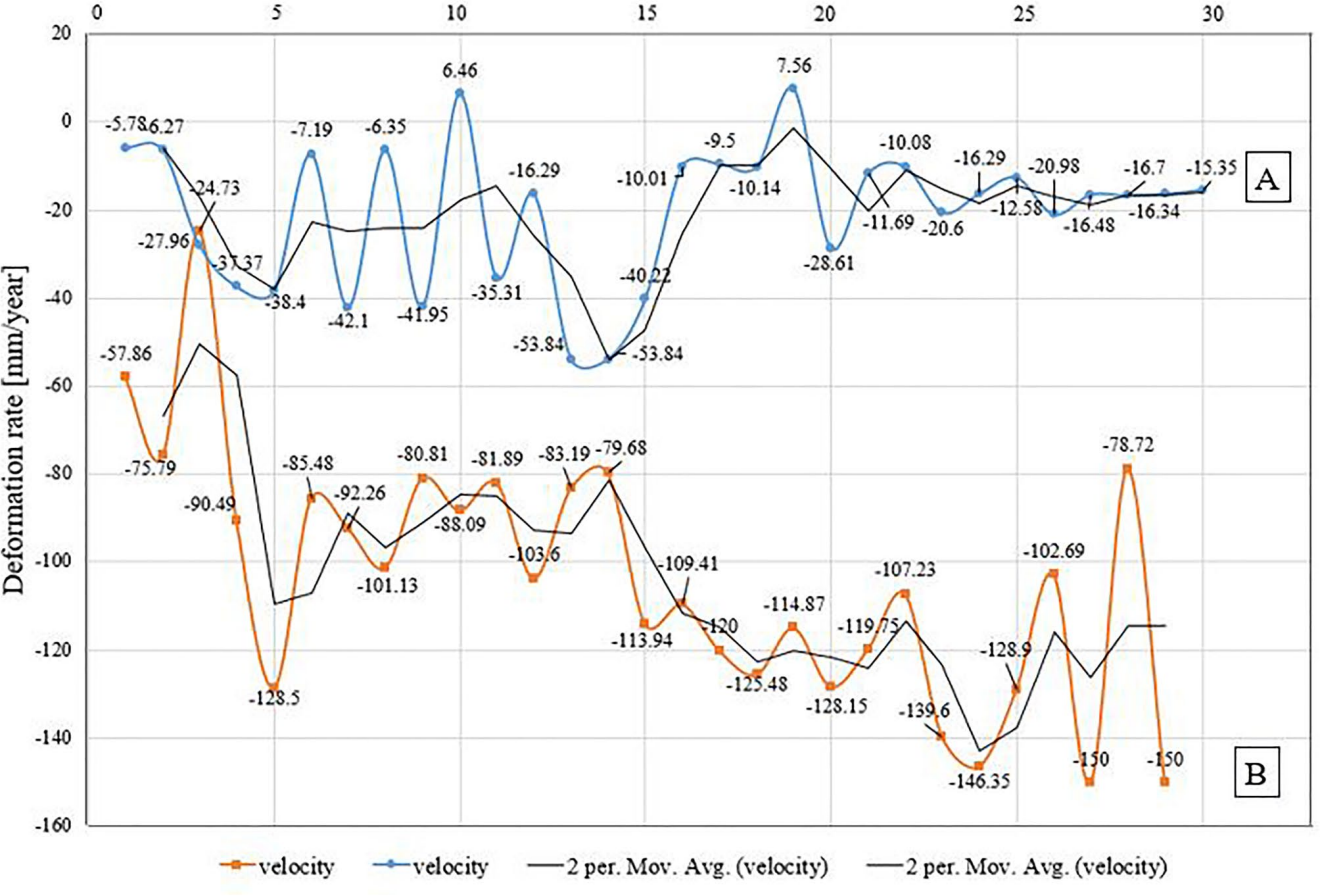
Deformation rate [mm/year] of Mach-A and Quetta-B region (Blue: Mach-A, Orange: Quetta-B). The rate of change in regional surface deformation (along the *y*-axis) was estimated by the coherent scattered PS points (along the *x*-axis).

Firstly, the results obtained from PS-InSAR and then combined with the Hypsometric Integral values. The areas of Quetta and Mach experienced the subsidence with a total velocity rate from − 4 to − 6 mm/year with the mature Hypsometric Integral values as in Fig. 17. The rate of uplift is nearly equal to the rate of erosion, as discussed in the results section. This region can experience a modest seismic forthcoming rate in the future and is susceptible to both erosion/uplifting with a velocity of more than − 6.0 mm/year. Chen also introduced this hypsometric technique of tectonic regimes in terms of stream-gradient. The hypsometric analysis was used to illustrate the relative activities sites in different tectonic regimes of Western Foothills^55^. Previous studies indicate the − 5.8 mm/year velocity in this region resulted from the tectonic processes and subsequently eroded towards subsidence^42^.

**Figure 18:**
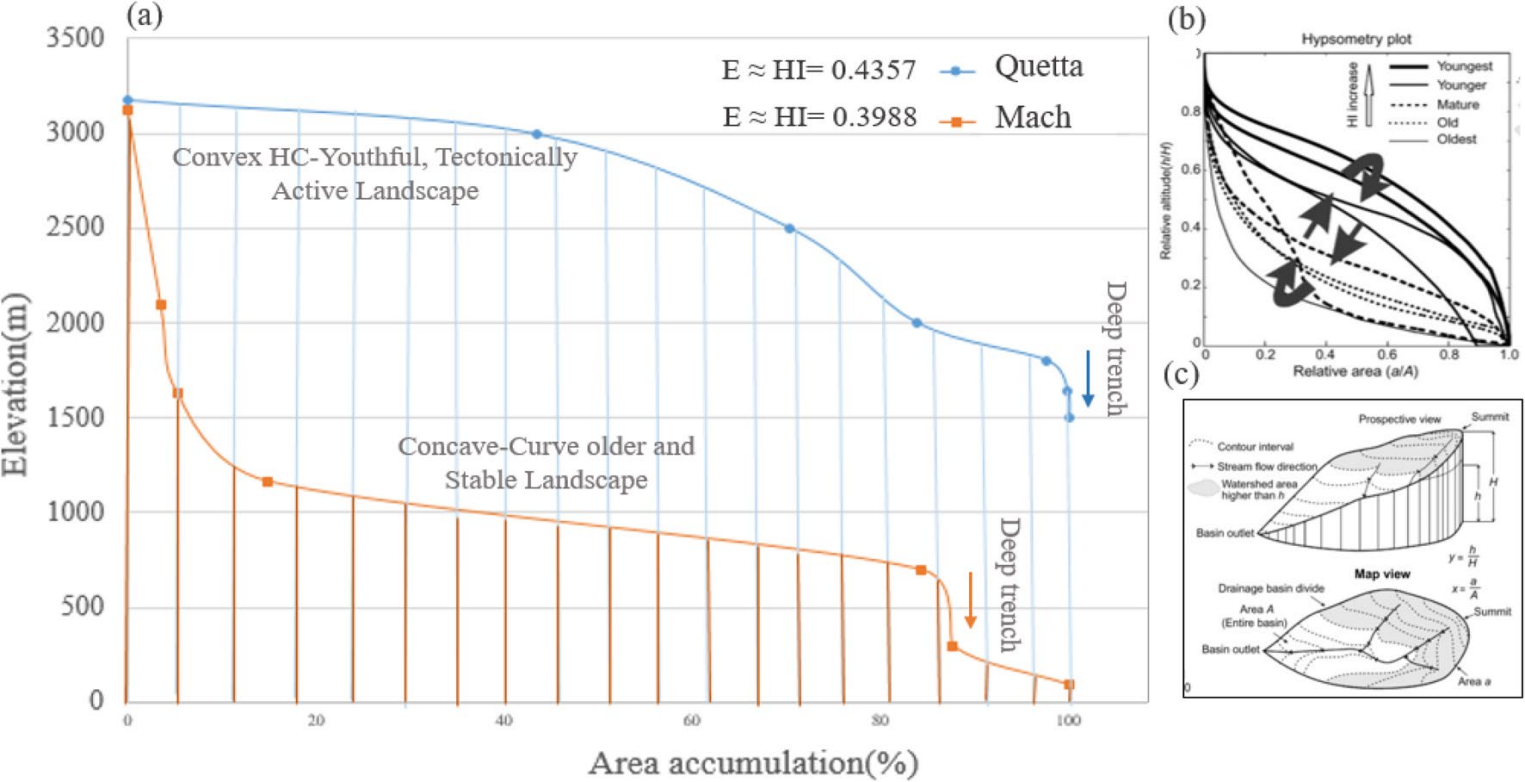
(**a**) Hypsometric curves (HC) of Earthquakes in the areas of Mach (orange) and Quetta (blue) cities. The convex and concave curves with the integral values of high (HI = 0.4257) describe the young stage, and the concave curve low (HI-0.398) defines an older and stable landscape. (**b**) Hypsometry plot defined by Strahler^37^, 1952 and (**c**) Geomorphic cycle by Perez-Pena^56^.

The InSAR observations form the topographic residuals, and according to the stratified tropospheric delay, the Chaman Fault shows ~ 8 mm/year relative motion. This segment of the Chaman Fault indicates the deformation accommodated by shallow fault creep. The maximum surface creeps rate of ~ 8 mm/year has been shown from 2004 to 2011^44^. The results obtained from the InSAR technique were compared with the measured geomorphic hypsometry integral curve values on the deflected streams networks. For this purpose, the hypsometry integral index value is defined by taking the area under the hypsometric curve and thus expresses the volume of a basin that has not been eroded. In Fig. 18a, hypsometric curve of Quetta consists of a steeper slope in the basin with the Integral values of HI_Mach_ = 0.43578. A deeper trench with the rapid fall of elevation observed at the end of the basin indicates an active tectonic landscape. The hypsometry curve of Mach was supposed to be steep at the beginning with the Integral values of HI_Mach_ = 0.398, then gentle in the middle of the basin, and a deeper trench with rapid fall in elevation was observed in the last. It shows that the basin is at an equilibrium stage where the rate of differential erosion is equal to the neo-tectonic level. Figure 18b shows the stage of equilibrium having with stable land characteristics as defined by Strahler^43^ and Fig. 18c shows Geomorphic cycle by Perez-Pena^56^ which describe the geomorphic properties of the site and their comparison. The changes in hypsometric describe prospectively.

**Figure 19:**
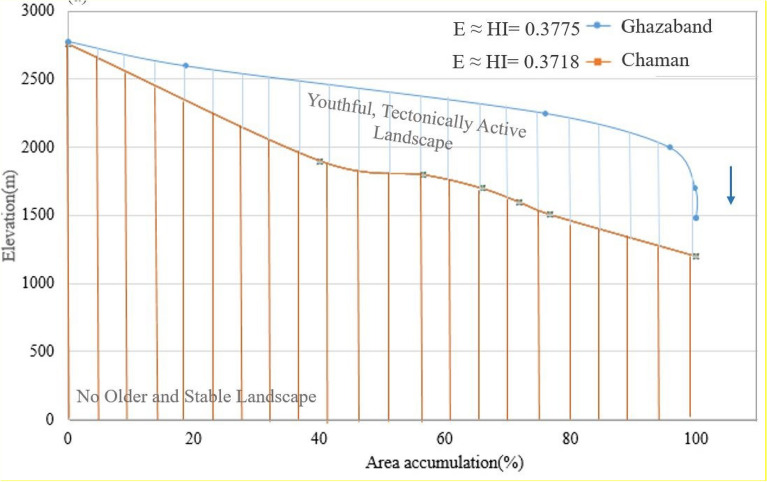
Hypsometric curves (HC) of Chaman fault (orange) and Ghazaband (blue) fault with S-shaped. The concave curves with the integral values of (HI = 0.3775, HI = 0.3718) describe the young tectonic landscape.

Hypsometry Integral values of Quetta, Mach, Chaman fault have been started towards the active tectonic regions with Integral values HI_Mach_ = 0.398 and HI_Quetta_ = 0.435 (0.35 ≤ HI ≤ 0.52); and HI ˃ 0.53 was the more susceptible class region with S and convex shape. These results were previously noted in the Neogene to Quaternary sedimentary Valley, which has proven a good technique for evaluating fault activity and related erosion processes^45^. The combination of PS-InSAR (remote sensing) and GIS (geographical Information system) results obtained through this study are evidence for active tectonics in this region. It is more likely to say that tectonic movement is responsible for causing surface deformation in the study areas. The Higher values of Hypsometry Integrals (HI) explain that it has not been eroded significantly. It is possibly formed by active tectonics and may recommend younger landscape characteristics, especially in the areas of Quetta, Chaman, and Ghazaband Faults. According to the given results of HI, we have assumed that the lower portion of the HI is perhaps related to tectonic uplifting along a Ghazaband fault, as shown in Fig. 19. Given results of the PS-InSAR and Hypsometric analysis indicates the seismic landscape vulnerability.

### Evidence of faults through fluvial incision deflection

**Figure 20:**
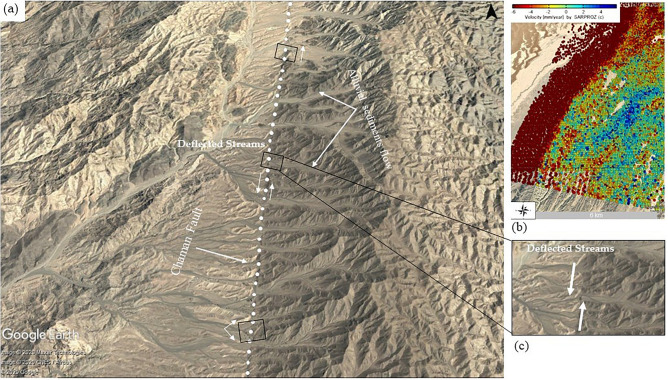
(**a**) Regional overview of Chaman fault, derived from Google Earth imagery. White circular lines are transform boundaries, deflected streams are shown in black rectangles networks and arrow bar indicates the reverse‐slip dislocation. (**b**) Time series surface deformation [mm/year] of Chaman with blue portion denotes movement towards radar LOS, respectively. (**c**) Zoom optical imagery view of Chaman fault location map with deflected dendritic stream network due to transform boundary. (© Basemap used from Google Earth platform).

**Figure 21:**
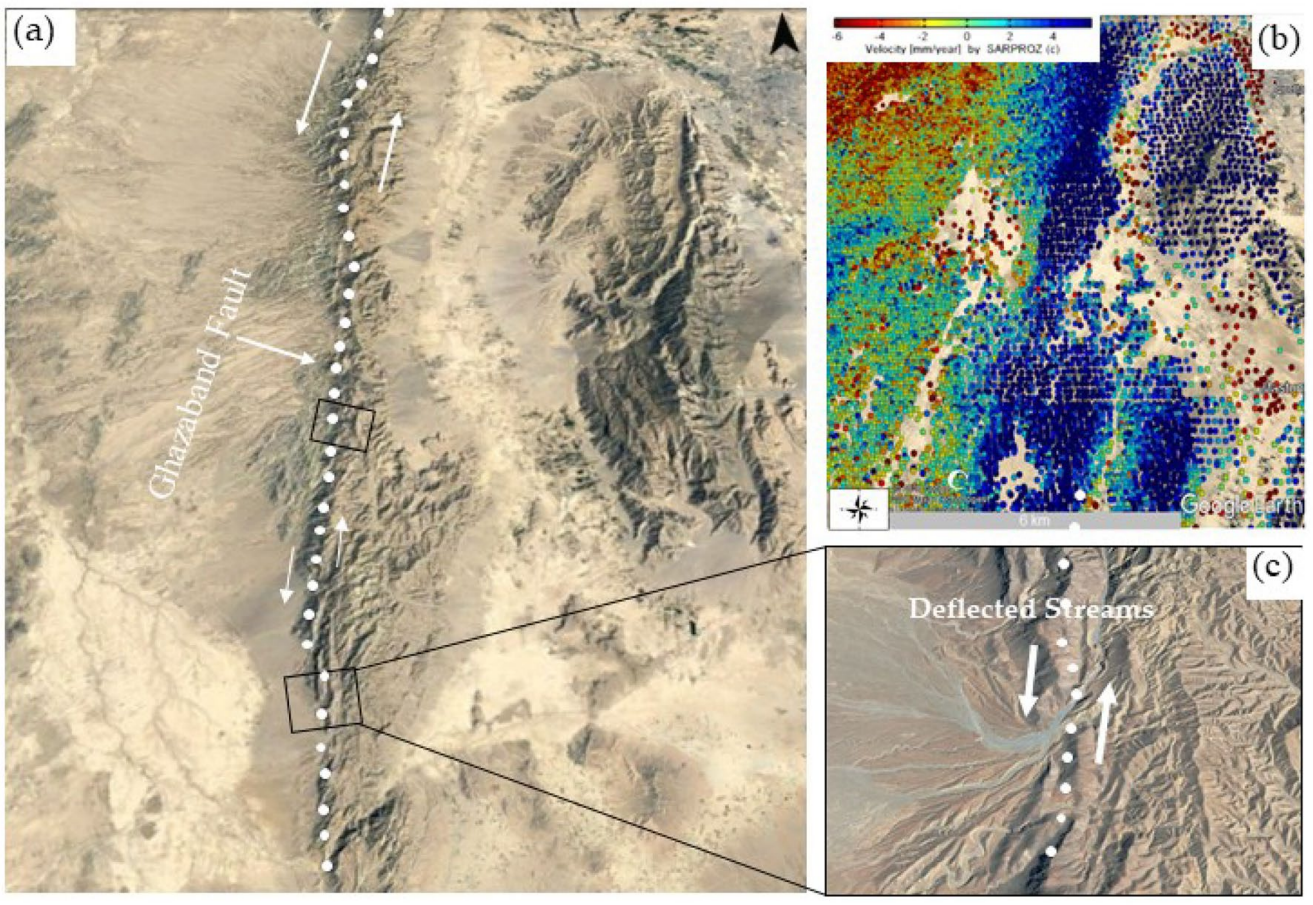
(**a**) Location map of the Ghazaband fault, derived from Google Earth imagery. White circular lines are strike‐slip dislocations, black circles are deflected stream networks and white arrow indicates the reverse‐slip dislocation. (**b**) Time series surface deformation [mm/year] of Ghazaband with blue portion denotes movement towards radar LOS, respectively. (**c**) Zoom optical imagery view of Ghazaband fault location map from Google Earth of the mountain range with deflected dendritic stream network (© Basemap used from Google Earth platform).

The evidence of Faults through fluvial incision deflection is shown in Figs. 20 and 21. These are the indication of the active element of the Chaman and Ghazaband fault system and these faults systems surround the populated cities of Quetta and Mach, Balochistan that provides an example of the interface between co-evolving and growing structures landscapes. Drainage patterns and landform/landscape examined in the Google earth system have been used to examine the lateral propagation of thrust in different regions. These systems along the fault lines are developing a semi-dendritic pattern with less defined and migrating or deflected stream patterns Fig. 20a,c. In the mapped area, the stream patterns are interchanging irrespective of the direction of strike-strip Chaman Fault.

The dominant control on this drainage pattern is the actively evolving structures associated with the other factors together with, bedrock lithology, land cover/land use and climate are constant in the study area and cannot explain the change in stream pattern. These streams commonly flow along with the lateral position of the fault. In these steeper terrain areas of Balochistan erosion rates are already determined using the Hypsometry Integral values (HI) and the fluvial incision has produced a variable degree of deflection. Deflected Fluvial networks along mountainsides with dendritic patterns along the active faults are also widespread in Fig. 21a,c of the Ghazaband Fault. In Fig. 21b, Time series surface deformation [mm/year] of Ghazaband with blue portion denotes movement towards radar LOS.

Their development and motion appeared to be organized by active tectonics, and therefore they hold the information about active landscape evolution. This fault line landscape evolution was also identified by the Time-series deformation approach by SAR Interferometry before the surrounded earthquake regions. This suggests the abandonment of the alluvial fan surface the Chaman fault has movement towards erosion in the time of 2018 and 2019 during the earthquakes in Mach and Quetta cities of Balochistan. Whereas the Ghazaband Fault region shows the total deformation velocity rate of 4 mm/year from the year 2018 to 2019 towards active uplifts and this dominant uplifting rate could be due to pioneer pressure increase in the Ghazaband fault and responsible for bringing out the Earthquake in the cities of Mach and Quetta.

## Conclusion

An earthquake of Mw 5.0 stokes on March 2019 in the Balochistan region. This earthquake caused significant surface deformation in Mach and Quetta, Balochistan. This study mainly focuses on the relative susceptibility and assessment of land deformation caused by Mw 5.0 earthquakes in Mach and Quetta, Balochistan, Pakistan. Moreover, combined approach of PS-InSAR and Hypsometric techniques has been used to indicate the future tendency and comparison of seismic sites. The objective of this research has been achieved by highlighting the surface displacement in tectonically active areas that are robustly influenced by deadly earthquake activities and comparison of surrounding seismic sites were also done. The estimated deformation compared the seismic vulnerability sites using the combined techniques of RS and GIS. The multi-temporal PS-InSAR method is applied for displacement analysis before and after the seismic activity in the study areas. The Mach region shows vertical displacement of 2 mm/day and − 9.5 mm/day during the co-seismic event. The PS time series analysis shows the displacement along the Line of Sight (LOS) in the pre-seismic event of Mach from − 10 to − 37 mm/year. Moreover, the landmass of Quetta city experienced two major earthquakes of Mw 4.2 in December 2018 and Mw 5.0 and in March 2019, respectively. Total vertical displacement of − 10 cm/day and − 12 cm/day was observed due to these earthquakes. Our research strongly observes that Mach and Quetta regions are between two lines known as the mature stage: class 1_moderate (0.35 ≤ HI ≤ 0.52); with integral HI_Mach_ = 0.398 and HI_Quetta_ = 0.435 with a modest seismic approaching rate in the future. Whereas, surrounding areas like Sibi, Harnai, Ziarat and Mastang are comparable less active than Mach, Quetta, Chaman and Ghazaband. These areas are also vulnerable to erosion/uplifting because of vertical displacement rates of more than ± 55 mm/year. However, class 2_high (HI ˃ 0.53) with the younger and more tectonically active region is surrounded by Chaman fault, which possesses future vulnerability regarding subsidence more than the existing velocity rate ~ 8 mm/year and Ghazaband fault showed unequalled uplifting, which is more than 5–6 mm/year rate. Chaman, Ghazaband, Quetta and Mach are found to be most seismic vulnerable and active regions where the probability of earthquakes are greater than other surrounding regions. Our study limitations were all the stations in the valley were not continuously operating and were recorded at an interval of two months or greater. Therefore, the ground-based GPS rates showed the deformation at around − 7.7 mm/year to identify the subsidence in the valley which is closer to the displacement rate of − 6.0 mm/year calculated in our study. However, the previous studies could identify the deformation up to − 5.1 mm/year rate. We can overcome this by placing more ground-based continuously working GPS stations to get the results more accurate. In the future, we will endeavour to investigate the pressure between the plates and assess the seismically vulnerable sites during surface rupture.

## Data Availability

The data and materials used in this article are available upon request by the correspondence author.

## Abbreviations

SAR, PS-InSAR: Synthetic aperture radar, persistent scatterer interferometric SAR
HI: Hypsometry integral
SRTM: Shuttle radar topography mission
DEM: Digital elevation model
HC: Hypsometry curves
ML: Machine learning
SVM: Support vector machine
RVS: Rapid visual screening
SNR: Signal-to-noise ratio
SPS: Sparse point selection
APS: Atmospheric phase screen
LS: Least square
ScanSAR: Scanning synthetic aperture radar
PSC: Permanent scatter candidates
h/H: Ratio of contour elevation height to basin’s maximum elevation height
a/A: Ratio of upper contour zone area to the total surface outlet area of the basin
LOS: Line of sight
mm/year: Millimeter per year
EP: Elevation profile
CMT: Centroid moment tensor
ESA: European space agency

## References

[1] Ivins ER, Dokka RK, Blom RG (2007). Post-glacial sediment load and subsidence in coastal Louisiana. Geophys. Res. Lett..

[2] Singh O, Sarangi A, Sharma MC (2008). Hypsometric integral estimation methods and its relevance on erosion status of North-Western Lesser Himalayan watersheds. Water Resour. Manag..

[3] Harirchian E (2020). A machine learning framework for assessing seismic hazard safety of reinforced concrete buildings. Appl. Sci..

[4] Harirchian E, Lahmer T (2020). Developing a hierarchical type-2 fuzzy logic model to improve rapid evaluation of earthquake hazard safety of existing buildings. Structures.

[5] Ali M, Shahzad MI, Nazeer M, Mahmood I, Zia I (2021). Estimation of surface deformation due to Pasni earthquake using RADAR interferometry. Geocarto Int..

[6] Garcia, M. E. *Modeling Vertical Deformation Associated with the 1931 Mach*, 1–22 (2006).

[7] Wang H (2022). Analysis and prediction of regional land subsidence with InSAR technology and machine learning algorithm. KSCE J. Civ. Eng..

[8] Gao M (2018). Regional land subsidence analysis in eastern Beijing Plain by InSAR time series and wavelet transforms. Remote Sens..

[9] Hu B, Chen J, Zhang X (2019). Monitoring the land subsidence area in a coastal urban area with InSAR and GNSS. Sensors (Switzerland).

[10] Ge, L., Rizos, C., Han, S. & Zebker, H. *Mining Subsidence Monitoring Using the Combined Insar and gps Approach—InSAR jest techniką po raz pierwszy zaproponowaną w 1974 roku, a po ponad dwóch dekadach rozwoju jest obecnie dobrze rozwinięta techniką i ma wiele zastosowań w mapowaniu topografii i określaniu zmian topograficznych*, 1–10 (2001).

[11] Murgante B (2014). Spatio-temporal Analysis of Earth’s Surface Deformation by GPS and InSAR Data BT—Computational Science and Its Applications—ICCSA 2014.

[12] Ramzan, U. & Hong, F. Combined GIS based spatial-temporal analysis using social media data of Wuhan, China. In *Eur. Conf. Educ. 2022 Off. Conf. Proc.,* 167–187. 10.22492/issn.2188-1162.2022.16 (2022).

[13] Chen B (2011). Spatial-temporal characteristics of land subsidence corresponding to dynamic groundwater funnel in Beijing Municipality, China. Chin. Geogr. Sci..

[14] Yeats, R. S. *et al*. Surface effects of the 16 March 1978 earthquake, Pakistan-Afghanistan border. *Geodyn. Pakistan Geol. Surv*, 359–361 (1979).

[15] Regard V (2005). Cumulative right-lateral fault slip rate across the Zagros-Makran transfer zone: Role of the Minab-Zendan fault system in accommodating Arabia-Eurasia convergence in southeast Iran. Geophys. J. Int..

[16] Kazmi., A. H. & Jam, M. *Geology and Tectonics* (1997).

[17] Jadoon IAK, Lawrence RD, Lillie RJ, McClay KR (1992). Balanced and retrodeformed geological cross-section from the frontal Sulaiman Lobe, Pakistan: Duplex development in thick strata along the western margin of the Indian Plate. Thrust Tectonics.

[18] Lawrence RD, Khan SH, Chaman TN (1992). Fault Pakistan-Afghanistan. Ann. Tectonicae.

[19] Philippe Davy PC (1988). Indentation tectonics in nature and experiment. 1. Experiments scaled for gravity. Bull. Geol. Inst. Univ. Uppsala.

[20] Nakata T, Otsuki K, Khan SH (1990). Active faults, stress field and plate motion along the Indo-Eurasian plate boundary. Tectonophysics.

[21] Jadoon I, Lawrence R, Lillie R (1994). Seismic data, geometry, evolution, and shortening in the active sulaiman fold-and-thrust belt of Pakistan, southwest of the Himalayas. Aapg Bull..

[22] Kopp C (2000). Structure of the Makran subduction zone from wide-angle and reflection seismic data. Tectonophysics.

[23] Yague-Martinez N (2016). Interferometric processing of sentinel-1 TOPS data. IEEE Trans. Geosci. Remote Sens..

[24] Hussain S (2021). Optimized landslide susceptibility mapping and modelling using PS-InSAR technique: A case study of Chitral valley, Northern Pakistan. Geocarto Int..

[25] Davidson, M. *et al.**Sentinel-1 Mission Overview*, Vol. 1, 1–4 (2010).

[26] Canaslan F, Ustun A (2012). Impact of perpendicular and temporal baseline characteristics on InSAR coherence maps. Remote Sens..

[27] Berardino P, Fornaro G, Lanari R, Sansosti E (2002). A new algorithm for surface deformation monitoring based on small baseline differential SAR interferograms. Geosci. Remote Sens. IEEE Trans..

[28] Qin Y, Perissin D, Bai J (2018). Investigations on the coregistration of sentinel-1 TOPS with the conventional cross-correlation technique. Remote Sens..

[29] Li Z, Bethel J (2008). Image coregistration in SAR interferometry. Proc Int. Arch. Photogramm. Remote Sens. Spat. Inf..

[30] Perissin D, Wang T (2012). Repeat-pass SAR interferometry with partially coherent targets. IEEE Trans. Geosci. Remote Sens..

[31] Bürgmann R, Rosen PA, Fielding EJ (2000). Synthetic aperture radar interferometry to measure earth’s surface topography and its deformation. Annu. Rev. Earth Planet. Sci..

[32] Hussain S, Hongxing S, Ali M, Ali M (2022). PS-InSAR based validated landslide susceptibility modelling: A case study of Ghizer valley, Northern Pakistan. Geocarto Int..

[33] Hussain S (2022). Optimized landslide susceptibility mapping and modelling using PS-InSAR technique: A case study of Chitral valley, Northern Pakistan. Geocarto Int..

[34] Devanthéry N, Crosetto M, Monserrat O, Cuevas-González M, Crippa B (2014). An approach to persistent scatterer interferometry. Remote Sens..

[35] Mora O, Lanari R, Mallorqui JJ, Berardino P, Sansosti E (2002). A new algorithm for monitoring localized deformation phenomena based on small baseline differential SAR interferograms. IEEE Int. Geosci. Remote Sens. Symp..

[36] Hurtrez JE, Sol C, Lucazeau F (1999). Effect of drainage area on hypsometry from an analysis of small-scale drainage basins in the siwalik hills (Central Nepal). Earth Surf. Process. Landforms.

[37] Strahler AN (1952). Hypsometric (area-altitude) analysis of erosional topography. GSA Bull..

[38] Ahmed F (2016). Hypsometric analysis of the Tuirini drainage basin: A geographic information system approach. Int. J. Geomatics Geosci..

[39] Pike RJ, Wilson SE (1971). Elevation-relief ratio, hypsometric integral, and geomorphic area-altitude analysis. GSA Bull..

[40] Holbrook J, Schumm SA (1999). Geomorphic and sedimentary response of rivers to tectonic deformation: A brief review and critique of a tool for recognizing subtle epeirogenic deformation in modern and ancient settings. Tectonophysics.

[41] Kusre BC (2013). Hypsometric analysis and watershed management of Diyung watershed in north eastern India. J. Geol. Soc. India.

[42] Huang J (2016). Study of subsidence and earthquake swarms in the western Pakistan. Remote Sens..

[43] Szeliga W, Bilham R, Kakar D, Lodi S (2012). Interseismic strain along the western boundary of the Indian subcontinent. J. Geophys. Res. Solid Earth.

[44] Fattahi H, Amelung F (2016). InSAR observations of strain accumulation and fault creep along the Chaman Fault system, Pakistan and Afghanistan. Geophys. Res. Lett..

[45] Pérez-Peña JV, Azañón JM, Booth-Rea G, Azor A, Delgado J (2009). Differentiating geology and tectonics using a spatial autocorrelation technique for the hypsometric integral. J. Geophys. Res. Earth Surf..

[46] Mahmood SA, Gloaguen R (2012). Appraisal of active tectonics in Hindu Kush: Insights from DEM derived geomorphic indices and drainage analysis. Geosci. Front..

[47] Sagintayev Z (2012). A remote sensing contribution to hydrologic modelling in arid and inaccessible watersheds, Pishin Lora basin, Pakistan. Hydrol. Process..

[48] Ambraseys N, Bilham R (2003). Earthquakes and associated deformation in Northern Baluchistan 1892–2001. Bull. Seismol. Soc. Am..

[49] Monalisa, Khwaja AA, Jassn MQ (2007). Seismic hazard assessment of the NW Himalayan fold-and-thrust belt, Pakistan, Using Probabilistic Approach. J. Earthq. Eng..

[50] Devanthéry N (2016). Deformation monitoring using persistent scatterer interferometry and sentinel-1 SAR data. Procedia Comput. Sci..

[51] Ali M, Shahzad M, Nazeer M, Kazmi J (2018). Estimation of surface deformation due to Pasni earthquake using SAR interferometry. Int. Arch. Photogramm. Remote Sens. Spatial Inf. Sci..

[52] Lu CH, Ni CF, Chang CP, Yen JY, Chuang RY (2018). Coherence difference analysis of sentinel-1 SAR interferogram to identify earthquake-induced disasters in urban areas. Remote Sens..

[53] Mahmood SA, Gloaguen R (2011). Analyzing spatial autocorrelation for the hypsometric integral to discriminate neotectonics and lithologies using DEMs and GIS. GISci. Remote Sens..

[54] Qureshi J, Mahmood SA, Masood A, Khalid P, DEM Kaukab IS (2019). and GIS-based hypsometric analysis to study tectonics and lithologies in southern Suleiman fold and thrust belt (Balochistan–Pakistan). Arab. J. Geosci..

[55] Chen YC, Sung Q, Cheng KY (2003). Along-strike variations of morphotectonic features in the Western Foothills of Taiwan: Tectonic implications based on stream-gradient and hypsometric analysis. Geomorphology..

[56] Pérez-Peña JV, Azañón JM, Azor A, Delgado J, González-Lodeiro F (2009). Spatial analysis of stream power using GIS: SLk anomaly maps. Earth Surf. Process. Landforms..

